# Serological Assays for Alveolar and Cystic Echinococcosis—A Comparative Multi-Test Study in Switzerland and Kyrgyzstan

**DOI:** 10.3390/pathogens11050518

**Published:** 2022-04-27

**Authors:** Philipp A. Kronenberg, Ansgar Deibel, Bruno Gottstein, Felix Grimm, Beat Müllhaupt, Cordula Meyer zu Schwabedissen, Sezdbek Aitbaev, Rakhatbek A. Omorov, Kubanychbek K. Abdykerimov, Gulnara Minbaeva, Jumagul Usubalieva, Mar Siles-Lucas, Paola Pepe, Laura Rinaldi, Markus Spiliotis, Junhua Wang, Norbert Müller, Paul R. Torgerson, Peter Deplazes

**Affiliations:** 1 Institute of Parasitology, Vetsuisse and Medical Faculty, University of Zurich, 8075 Zurich, Switzerland; felix.grimm@uzh.ch; 2 Graduate School for Cellular and Biomedical Sciences, University of Bern, 3012 Bern, Switzerland; 3 Clinics of Hepatology and Gastroenterology, University Hospital Zurich, 8091 Zurich, Switzerland; rudolfansgar.deibel@usz.ch (A.D.); beat.muellhaupt@usz.ch (B.M.); cordula.meyerzuschwabedissen@usz.ch (C.M.z.S.); 4 Laboratory of Parasitology, Institute for Infectious Diseases, Medical Faculty, University of Bern, 3001 Bern, Switzerland; bruno.gottstein@ifik.unibe.ch (B.G.); markus.spiliotis@ifik.unibe.ch (M.S.); junhua.wang@ifik.unibe.ch (J.W.); 5 City Clinical Hospital #1, Surgical Department, Faculty of Surgery of the Kyrgyz State Medical Academy, Bishkek 720054, Kyrgyzstan; siezbekaitbaev@gmail.com (S.A.); rahatbekomorov@mail.ru (R.A.O.); 6 Section of Epidemiology, Vetsuisse Faculty, University of Zurich, 8057 Zurich, Switzerland; kubanychbek.abdykerimov@uzh.ch (K.K.A.); paul.torgerson@uzh.ch (P.R.T.); 7 Life Science Zürich Graduate School, University of Zürich, 8057 Zurich, Switzerland; 8 Government Sanito-Epidemiology Unit, Kyrgyz Ministry of Health, Bishkek 720033, Kyrgyzstan; minbaevag-mz@mail.ru (G.M.); jumagul2006@mail.ru (J.U.); 9 Instituto de Recursos Naturales y Agrobiologia (IRNASA-CSIC), 37008 Salamanca, Spain; mmar.siles@irnasa.csic.es; 10 Unit of Parasitology and Parasitic Diseases, Department of Veterinary Medicine and Animal Production, University of Naples Federico II, 80138 Naples, Italy; paola.pepe@unina.it (P.P.); lrinaldi@unina.it (L.R.); 11 Institute of Parasitology, Vetsuisse Faculty, University of Bern, 3012 Bern, Switzerland; norbert.mueller@vetsuisse.unibe.ch

**Keywords:** *Echinococcus multilocularis*, *Echinococcus granulosus sensu lato*, diagnosis, serology, antigens, antibodies, ELISA, Western blot

## Abstract

Both alveolar (AE) and cystic echinococcosis (CE) are lacking pathognomonic clinical signs; consequently imaging technologies and serology remain the main pillars for diagnosis. The present study included 100 confirmed treatment-naïve AE and 64 CE patients that were diagnosed in Switzerland or Kyrgyzstan. Overall, 10 native *Echinococcus* spp. antigens, 3 recombinant antigens, and 4 commercial assays were comparatively evaluated. All native *E. multilocularis* antigens were produced in duplicates with a European and a Kyrgyz isolate and showed identical test values for the diagnosis of AE and CE. Native antigens and three commercial tests showed high diagnostic sensitivities (Se: 86–96%) and specificities (Sp: 96–99%) for the diagnosis of AE and CE in Swiss patients. In Kyrgyz patients, values of sensitivities and specificities were 10–20% lower as compared to the Swiss patients’ findings. For the sero-diagnosis of AE in Kyrgyzstan, a test-combination of an *E. multilocularis* protoscolex antigen and the recombinant antigen Em95 appears to be the most suitable test strategy (Se: 98%, Sp: 87%). For the diagnosis of CE in both countries, test performances were hampered by major cross-reactions with AE patients and other parasitic diseases as well as by limited diagnostic sensitivities (93% in Switzerland and 76% in Kyrgyzstan, respectively).

## 1. Introduction

Alveolar and cystic echinococcosis (AE/CE) are among the most lethal parasitic diseases and are nevertheless classified as neglected zoonoses. AE is caused by the larval stage of *Echinococcus multilocularis,* which resides in the small intestines of predominantly foxes, dogs and other wild canids. After oral ingestion of eggs by intermediate hosts like rodents or dead-end hosts such as humans, the subsequently developing and progressively growing metacestode leads to a tumor-like pathology and is usually fatal if not appropriately treated [1,2]. Moreover, *Echinococcus granulosus sensu lato* (*s.l*.) causes CE, leading to space-occupying, fluid-filled, mostly single cysts primarily located in the liver and lungs of infected persons. The natural life-cycle of *E. granulosus s.l.* includes canids as definitive hosts and in particular sheep and other ruminants as intermediate hosts [3]. While *E. multilocularis* is confined to the Northern hemisphere, *E. granulosus s.l.* has a worldwide distribution [4]. Both parasites are responsible for extensive human morbidity with an estimated global 18,400 new AE and 188,000 CE patients, and approximately 19,000 people succumb to both diseases per year [5]. Treatment options include primarily surgery and medication with benzimidazoles, thereof mainly albendazole [6]. 

In Europe and North America, AE is considered an emerging disease with increasing incidences over the last two decades. Around 200–300 new AE patients are expected to be diagnosed in central Europe every year, where regional annual incidences are ranging between 0 and 8.1/100,000 inhabitants [7,8]. The overall yearly AE incidence in Switzerland has significantly increased from 0.1 between 1993–2000 to 0.26 in 2001–2005 [9] and persisted relatively high in the last decade [4]. For CE, the annual surgical incidence ranges between 2.3/100,000 inhabitants and 18.0/100,000 in highly endemic countries [10]. As an example for CE in Europe, around 900 new hospitalized CE patients are expected every year in Italy [11]. A recent study has found evidence that the prevalence of CE is unknown or underestimated in Europe and continues to remain a public health issue on its way to disease elimination postulated by the World Health Organization (WHO) [12].

Large parts of Asia including Russia, Turkey, Central Asia, and Japan represent extended endemic areas for AE. Countries with a high number of AE patients are furthermore China and Kyrgyzstan with prevalences as high as 15% in some villages in China [13] and 4% in Kyrgyzstan [14]. From 2014 to 2016, the country-level crude surgical incidence in Kyrgyzstan was 3.02/100,000 inhabitants per year for AE, considered to be the highest global country-wide incidence for this disease. In the same study, considering hotspots on the local community level, yearly incidences as high as 246/100,000 inhabitants for AE were observed. In Kyrgyzstan, the annual surgical incidence for CE from 2014 to 2016 was 13.1/100,000 inhabitants per year with local community-level hotspots as high as 176/100,000 [15]. Both CE and AE patients have significantly increased in Kyrgyzstan over the last decade [16]. The age distribution of Kyrgyz AE patients differs from Swiss patients, with a mean average age of 32 years in Kyrgyzstan [15] compared to 54 years in Switzerland [17].

The diagnosis of echinococcosis is based on a combined approach primarily upon imaging technologies, complemented by serology, histopathology, and molecular methods. Both AE and CE are lacking specific pathognomonic clinical signs; therefore, imaging technologies supported by serology are the main pillars for a clinical-based diagnosis of echinococcosis [2,6]. Over the last decades, a vast number of serological assays for the detection of antibodies against a variety of *Echinococcus* spp. antigens have been developed. These antigens have been successfully used in clinical as well as in sero-epidemiological studies [18]. In recent years, technologies based on ELISA, Western blot, and lateral flow assays have been applied in commercialized or laboratory validated tests. Interestingly, so far all native and even recombinant antigens of *E. granulosus s.l.* have shown relatively high cross-reactions with antibodies from AE patients [19,20]. Conversely, antigens of *E. multilocularis*, especially Em2, Em2G11, as well as the recombinant antigen Em18, exhibit a relatively high specificity in many assays for the diagnosis of AE, approaching >99% when using locally adapted and optimized cut-off values [20,21]. Nevertheless, the early and also the subclinical diagnosis of AE seems still to be challenging, especially in specific geographical areas such as Kyrgyzstan, where a study reported only low sensitivities of serological assays [14]. In the same context, an interesting observation was noted: the mean size of lesions in ELISA- or Western blot sero-positive patients (N = 27) was 46 mm, which was significantly larger than the mean size of 11 mm for lesions from the sero-negative patients (N = 22) [14]. Similar test characteristics were also observed in neighboring China [22]. Consequently, and according to WHO recommendations, there is a need to improve the early detection of both CE and AE, especially in low-resource settings like Kyrgyzstan [23].

The sero-diagnosis of AE is one of the best-developed areas in medical helminthology. Both native antigens of *E. multilocularis* and *E. granulosus s.l.* and several recombinant antigens have been evaluated in a variety of studies [18]. Native *E. multilocularis* metacestode crude antigens originating from experimentally infected rodents show high sensitivities in diagnostic assays but are contaminated with host proteins. Therefore, those antigens are increasing the cut-off values of the assays. Furthermore, cross-reacting parasite epitopes are resulting in high cross-reactions, especially for patients with other helminthic infections. To overcome the lack of specificity, a native affinity-purified antigen fraction named Em2 has been developed [24]. Em2 was purified by absorbing the cross-reacting epitopes with antibodies directed against *E. granulosus s.l.* metacestode antigens. Later, the Em2G11 antigen fraction was affinity-purified with the highly Em2-specific monoclonal antibody Em2G11. Both Em2 and Em2G11 antigens showed identical diagnostic properties in a comparative study [25]. Em2G11 is a mucin-type glycoprotein, and it is part of the laminated layer that surrounds and protects the parasite inside the host [26]. An advantage of this antigen is the high specificity, especially regarding cross-reactions with CE patients [20,21]. 

Another approach to produce more stage-specific native antigens was the purification of protoscolices from the metacestode material of infected rodents [27]. This approach was further optimised by affinity purification (magnetic beads based) of the protoscolex fraction by eliminating the numerous small vesical cysts (EmP antigen). This step resulted in a high sensitivity of >99% for AE patients and a specificity of 74% in patients with various parasitic diseases [20]. Another approach to produce metacestode antigens without much host tissue contamination was the in vitro cultivation of metacestode material from infected rodents. Vesicle fluid from in vitro-cultivated *E. multilocularis* vesicles improved the antigen purity and enabled the production of more defined crude antigens with excellent diagnostic properties. The use of vesicle fluid (EmVF) and subsequentially the development of an EmVF-immunoblot has been evaluated in different publications and was described as superior compared to other native antigens of *Echinococcus* species [28]. Especially the specificity in the EmVF-immunoblot for AE diagnostics could be improved with 50% cross-reactions in CE patients and no cross-reactivity with other parasites [29].

So far two promising recombinant antigens named recEm18 (II/3-10) and recEm95 have been developed and used as serological reagents. The recEm18 antigen was used for decades in specialised laboratories and represents one of the antigens of the commercialised Em2+ antigen assay [21]. The antigen recEm18 shows striking homologies compared to the earlier-discovered II/3 and II/3-10 antigens. Most likely the diagnostic epitope of recEm18 is part of the bigger II/3-10 antigen, by covering overlapping segments in the same gene and proteins [30]. The recEm18 antigen has furthermore proved its use in the serological follow up of AE patients, especially for assessing radical resections of surgical interventions and to evaluate a possible curative course of AE after long-term chemotherapy or lesion activity [28,31,32,33].

The recEm95 antigen is the analogue to the recEg95 antigen, which has been demonstrated to be a highly effective ovine vaccine in New Zealand, Australia, and Argentina [34]. It was initially published by Gauci et al., (2022) [35] who demonstrated that the EM95 recombinant protein can be used to induce significant levels of protection against challenge infection with *E. multilocularis* eggs in mice. The recEm95 antigen, so far, proved diagnostic efficiency in ELISA within veterinary parasitology, where it exhibited the best serodiagnostic operating characteristics in AE-affected dogs when compared to multiple other *E. multilocularis* antigens [36].

Serological assays for the diagnosis of CE include native antigens of *E. granulosus s.l.*, such as cyst fluid (EgCF), protoscolex antigen (EgP), antigen B (AgB), and antigen 5 [18]. Cyst fluid of *E. granulosus s.l*. from a variety of intermediate hosts usually shows sensitivities of 65–100% for the serological diagnosis of CE patients, depending on the study groups (age, geographical origin), treatment options, cyst staging and activity. Interestingly, *E. granulosus s.l.* cyst fluid antigen (EgCF) demonstrated a very high sensitivity of >99% for the diagnosis of AE in a study by Schweiger and colleagues (2012), but consequently had a low specificity of 71% for various parasitic diseases [20]. Similar values of sensitivities ranging from 69–97% could be observed in antigens derived from *E. granulosus s.l.* protoscolices (EgP antigen), which can be easily isolated from fertile cysts from intermediate hosts [19]. In this context, some diagnostic assays for the diagnosis of CE are significantly influenced by cyst activity, size, number, localization, and treatment before serum collection [37]. A high number of available tests for the diagnosis of CE are based on detecting antibodies against crude antigens of *E. granulosus s.l.* and are tagged by relatively poor specificity and limited sensitivity [18,19]. Furthermore, several recombinant antigens have been developed over the years. Until now, recombinant antigens deriving from genes of antigen B seem to have the most potential in the serological diagnosis of CE. In particular, a tandem repeat of the antigen AgB2, named 2B2, had a superior sensitivity of 88% in the serological diagnosis of 186 confirmed CE patients, compared to standard assays like EgCF-ELISA (Se: 83%) and indirect haemagglutination, IHA (Se: 35%) [38]. 

For the serological diagnosis of AE and CE, a variety of assays are commercially available [18,39,40]. For the serological differentiation between CE and AE, two Western blot assays are commercially available. The Echinococcus-LD-BIO™ Western blot (LDBIO Diagnostics, Lyon, France) uses a whole larval extract of *E. multilocularis* for the species differentiation of *Echinococcus* spp. In comparison to that, the Echinococcus-EUROLINE™-Western blot (EUROIMMUN AG, Lübeck, Germany, test applied in this study) uses *E. multilocularis* vesicle fluid and three recombinant antigens (recEgAgB, recEm18, recEm95). Both assays have been evaluated in different studies, claiming superior sensitivities of 92–98% and specificities up to 100% compared to standard assays [41,42,43].

To conclude, in laboratories specialized for the serology of human echinococcosis, a combination of several tests/antigens is applied with mostly satisfying results. Major drawbacks for assays for the diagnosis of CE and AE are cross-reactions with other parasites. Particularly CE patients have major cross-reactions in serological tests for the diagnosis of AE and other helminths and vice versa. The goal of this study was the evaluation and comparison of 17 diagnostic assays for the detection of specific serum antibodies in human alveolar and cystic echinococcosis for use in a clinical context or for population screenings in endemic regions. 

## 2. Material and Methods 

### 2.1. Serum Samples of AE and CE Patients

Serum samples of treatment-naïve patients diagnosed with AE (n = 60) and CE (n = 41) at the University Hospital of Zurich, Switzerland (Echinococcosis Cohort Study) were included in this study, before surgical resection and/or the initiation of chemotherapy with benzimidazoles. Kyrgyz serum samples of treatment-naïve patients included 40 AE and 23 CE patients that were diagnosed and had surgery at City Clinical Hospital #1 in Bishkek, Kyrgyzstan. Case inclusion criteria in both countries were solely based on positive PCR results [44,45] or immunohistochemical-stainings with monoclonal antibodies of resection specimens or biopsies [46]. Lesions were classified according to WHO standards [6] and clinical data was noted. 

Patients diagnosed with CE in Switzerland were predominantly originating from countries in South Europe (39/41; Italy, Turkey, Macedonia, Kosovo, Albania, Montenegro, Portugal). Two CE patients were originally from Switzerland and never lived abroad. Cysts were localized in the liver (37 patients), liver and abdomen (2), spina iliaca (1), and lower leg/tibia (1). Most AE patients diagnosed in Switzerland were of Swiss nationality (51/60). Nine patients had immigrated to Switzerland more than 20 years previously. The liver was involved in all 60 AE patients from Switzerland, and 11 patients additionally showed involvement of the lung (5), brain (2), lung and brain (1), lumbar vertebrae (1), abdomen (1), and peritoneum (1). 

Patients diagnosed with AE in Kyrgyzstan originated from the following oblasts/regions; Issyk-Kul (5), Naryn (10), Osh (23), Jalal-Abad (1), and Chuy (1). The liver was involved in all 40 AE patients from Kyrgyzstan with an additional involvement of the lungs in one patient. Kyrgyz CE patients originated from Naryn (5), Osh (1), Jalal-Abad (2), Chuy (11), Batken (2), and Talas (2). Cysts were localized in the liver (20 patients), lung (2), and liver and kidney (1). 

Further patient’s characteristics are shown in Table 1. (Gender, age, WHO case definition and respective case classification/staging, lesion size, total immunoglobulin E values (IgE), and lymphocyte count). Total IgE values and lymphocyte count of Swiss echinococcosis patients were included for a possible support of the clinical diagnosis. Absolute and relative lymphocyte count was determined by using the Sysmex XN-20 (Sysmex), a fluorescence–flow-cytometry system with automated cell counting. Total IgE was measured by using the immunoassay kits ImmunoCAP Total IgE (ThermoFisher) and Optilite Immunoglobulin E (Optilite). 

To evaluate diagnostic strategies for the subclinical diagnosis of AE, 10 Swiss patients were included with lesion sizes below 2.5 cm. All 10 patients were women with an average age of 52 years. In six of those 10 patients, small AE lesions in the liver were found during routine diagnostics or surgeries as incidental findings. Three patients were classified as P1N0M0, two as P2N0M0, one as P1N1M0, and four as P1N0M1. These four patients had additional small metastases in the lungs (2), in the lumbar region (1), and in the vertebrae (1). One patient had a relapse 10.8 years after surgical resection. Three patients were evaluated with a positron emission tomography (PET) scan, but only one patient showed an increase of the 18F-FDG signal. Three patients showed elevated total IgE values (IgE values > 100 kU/L) and two patients had lymphopenia (<1.50 × 10^3^/µL). In 8 out of 10 patients, the serology for recEm18 was positive.

**Table 1:** Characteristics of patients with alveolar (AE) and cystic echinococcosis (CE).

Patient’s Characteristics	Swiss AE	Kyrgyz AE	Swiss CE	Kyrgyz CE
Number of patients	**N = 60**	**N = 40**	**N = 41**	**N = 23**
Males	22	18	22	11
Females	38	22	19	12
Average age males	58 (18–79)	32 (9–65)	38 (20–66)	38 (23–73)
Average age females	54 (21–72)	28 (7–57)	41 (12–60)	36 (17–64)
WHO classification/staging *	P1N0M0: 18	P1N0M0: 0	CE1: 11	CE1: 12
	P2N0M0: 11	P2N0M0: 6	CE2: 10	CE2: 7
	P3N0M0: 5	P3N0M0: 6	CE3: 8	CE3: 3
	P4N0M0: 1	P4N0M0: 2	CE4: 3	CE4: 1
	PXN1MX: 18	PXN1MX: 6	CE5: 2	CE5: 0
AE & CE: M1/disseminated	PXNXM1: 11	PXNXM1: 1	4	0
N/A: not available	N/A: 0	N/A: 20	N/A: 3	N/A: 0
**Lesion size (cm)**				
0.5–2.5	10	1	1	0
2.5–5.0	18	2	12	3
5.0–7.5	11	9	8	8
7.5–10.0	6	5	7	9
>10.0	15	7	8	3
N/A	0	16	5	0
**Total IgE**				
pre-treatment data avaliable	56/60	N/A	37/41	N/A
average IgE values	1262 kU/L	N/A	493 kU/L	N/A
median IgE values	159 kU/L	N/A	110 kU/L	N/A
elevated IgE values (>100 kU/L)	32 (57%)	N/A	20 (54%)	N/A
**Lymphocytes**				
pre-treatment data avaliable	56/60	N/A	37/41	N/A
average lymphocytes ×10^3^/µL	1.69	N/A	1.98	N/A
median lymphocytes ×10^3^/µL	1.73	N/A	1.85	N/A
Lymphopenia (<1.50 × 10^3^/µL)	21 (38%)	N/A	7 (19%)	N/A
**Elevated IgE and/or lymphopenia**	40 (71%)	N/A	24 (65%)	N/A

* according to Brunetti, 2010. [6], N/A: Not available.

### 2.2. Serum Samples for Evaluating Cut-off Values and Cross-Reactions

Human serum samples that were used for assessing test specificities and cross-reactions were obtained from the anonymized serum collection of the Institute of Parasitology in Zurich and Bern. The parasitic infections were confirmed histologically, clinically or parasitologically, involving the following parasites (16 serum samples each): *Schistosoma* spp., *Toxocara* spp., *Strongyloides stercoralis*, *Entamoeba histolytica*, *Trichinella* spp., *Taenia solium*, *Fasciola hepatica, Ascaris lumbricoides*, and filarial species. Serum samples of 68 healthy Swiss blood donors and 68 ultrasound-negative (US negative) residents from Kyrgyzstan from a previous study [14] were included to calculate country-specific cut-off values. Serum samples (n = 38) of patients with non-parasitic liver lesion (NPLL) were assessed to calculate clinical specificities of diagnostic tests as previously described by Schweiger et al. (2012). These patients were clinically and/or pathologically diagnosed with non-*Echinococcus* liver cysts, neoplasia, cholangitis, etc. [20].

### 2.3. Parasite Isolates 

#### 2.3.1. *E. multilocularis* European Isolate J2012

*Echinococcus multilocularis* metacestode tissue was obtained upon necropsy of a male long-tailed macaque that died of AE in a zoo in Germany in 2012. The isolate J2012 belongs to the European haplotype E4 of *E. multilocularis*, based on sequenced parts of the mitochondrial genome (cob, nad2, cox1). This European *E. multilocularis* isolate was initially described by Nakao et al. (2009) [47] and was later found in France and Belgium. Molecular analysis was performed as described by Alvarez–Rojas et al. (2020) [45].

#### 2.3.2. *E. multilocularis* Asian Isolate AT17

*Echinococcus multilocularis* metacestode tissue was isolated from the liver of a 17-year-old Kyrgyz male patient, suffering from AE in 2017. The infected part of the liver was resected during a curative surgical intervention. The isolate AT17 (Asian Haplotype A17) is one nucleotide different from the most common haplotype A2 of *E. multilocularis* in Kyrgyzstan (out of 3558 base pairs of the mitochondrial genome) [45]. For both isolates, the metacestode containing parts of the liver were washed in sterile phosphate-buffered saline (PBS) and stored for 3 days in 2 × PSF/PBS at 4 °C (A5955 Sigma, Antibiotic Antimycotic Solution)**.** The infected parts of the liver were subsequently minced into small pieces and pushed through a metal sieve (0.5 mm) with a syringe plunger. The sediment was injected intraperitoneally into gerbils. The life cycle of the isolates was maintained via repetitive intraperitoneal transplantation as described elsewhere [48]. The in vitro cultivation of *E. multilocularis* vesicles for antigen preparation was performed in principal as described by Laurimäe et al., (2020) [49], which was adapted from Spiliotis et al., (2009) [50].

#### 2.3.3. *E. granulosus sensu stricto* (*s.s.*) Sheep Isolate

One fertile cyst (10 × 10 cm) of an infected sheep liver was collected at an abattoir in Salerno, Southern Italy. The cox1 gene of the protoscolices was genetically sequenced according to Alvarez–Rojas et al. (2020) [45] and confirmed as *Echinococcus granulosus s.s.* G1 genotype. All native antigens of *E. granulosus s.s.* that were evaluated in this study originated from sheep liver single cysts and belong to the G1 genotype. 

### 2.4. Antigens & Commercial Tests

For a better overview and introduction into the results section, all antigens and commercial tests are summarized in Table 2.

(Results Section 3.1).

#### 2.4.1. In Vitro-Produced Vesicle Crude Antigens of *E. multilocularis* & *E. granulosus s.s.*

Vesicles of *E. multilocularis* were cultivated according to Laurimäe et al., (2020) [49], which was adapted from Spiliotis et al., (2009) [50]. After 6 months of in vitro cultivation, the vesicles of *E. multilocularis* were harvested and washed 5× in PBS. The vesicles were then poured and retained in a sterile metal sieve (0.5 mm) and further drained into a triangle-shaped 50 mL basin. For the production of *E. multilocularis* vesicle fluid (EmVF), the vesicles were squeezed through a sterile metal sieve (0.5 mm) with a syringe plunger. The vesicle fluid was further centrifuged at 16,000× *g* for 10 min at 4 °C. The supernatant was dialyzed against PBS for 24 h at 4 °C (SnakeSkin™ Dialysis Tubing, 10 KDa MWCO, ThermoFisher Scientific, Reinach, Switzerland). 

For the production of *E. multilocularis* vesicle crude antigen (EmVC)**,** the vesicles were washed 5× in PBS, subsequently drained and then cut in half with a scalpel blade in a 50 mL basin. The empty vesicles were additionally washed 5× in PBS. Four steel beads (3 mm) were added to the divided vesicles floating in PBS and mechanically disrupted in a tissue lyser for 1 min in 2 mL Eppendorf tubes (Qiagen TissueLyser II, 30 cycles per second). The solution was subsequently frozen on dry ice and thawed/frozen three times in a row. The vesicles were further processed with ultrasonication steps (10 cyles, 5s). The lysate was centrifuged at 16,000× *g* for 10 min at 4 °C. The supernatant was finally dialyzed against PBS for 24 h at 4 °C as described above.

For the production of *E. granulosus s.s.* vesicle crude antigen (EgVC), one fertile cyst (10 × 10 cm) of an infected sheep liver was collected and stored at 4 °C until further use. The cyst was afterwards washed with PBS, and the injection site was disinfected three times in a row with 70% ethanol followed by 10% povidone–iodine solution. The cyst was flushed with sterile PBS in a 60 mL syringe under sterile conditions and the cyst fluid containing germinal cells, protoscolices, and brood capsules was aspirated. The aspirated cyst fluid was further centrifuged at 250× *g* for 5 min. The protoscolices, germinal cells, and brood capsules in the sediment were then cultured for 6 months exactly as described for *E. multilocularis* until the evolved *E. granulosus s.s.* vesicles reached sizes of 4–10 mm. The further production of EgVC was performed as already described for *E. multilocularis*. All antigens were stored at minus 80 °C until further use. 

#### 2.4.2. Antigen on-Plate Purification with mAb Em2G11 and mAb EmG3

We have evaluated two monoclonal antibodies (mAb) for the on-plate purification of the corresponding antigen from in vitro-cultivated vesicles of *E. multilocularis* and *E. granulosus s.l.* The mAb EmG3 originates from the same fusion as the mAb Em2G11, but it is also directed against *E. granulosus s.l.* and *E. vogeli*, but does not react with other taeniid species or helminths. Both mAb EmG3 and Em2G11 bind to the native antigen Em2 of *E. multilocularis* and the laminated layer [25,46]. In this study the mAbs were coated on ELISA plates at 5 μg/mL as described for antigens (Material and Methods 2.4.5). After blocking, either *E. multilocularis* vesicle crude antigen (EmVC) or *E. granulosus* s.s. vesicle crude antigen (EgVC) was added according to previous titration studies for 1 h, washed 3× with PBS-tween 0.05%, followed by adding the patient’s sera, anti-human conjugate and substrate for the serological diagnosis of AE (EmVC antigen) and CE (EgVC antigen). 

#### 2.4.3. recEm95 Antigen

The recEm95 antigen was produced in principle as described by Gauci et al., (2002, 2011) [35,51]. The corresponding Em95 sequence was cloned into an EcoR1 linearized modified pET-151D vector by Hot Fusion Cloning as described by Fu et al., (2014) [52] for subsequent conventional protein expression. After protein expression, bacterial cell lysates were sedimented to remove any remaining cell debris. Supernatants were then processed upon affinity purification of recEm95 with PureCube His Affinity MagBeads Ni-NTA (Cube Biotech, Monheim am Rhein, Germany). The His-tagged recEm95 was then eluted in LEW buffer (lysis equilibration wash buffer) containing 200 mM imidazole. The protein concentration was determined by the Bradford method. 

#### 2.4.4. Commercial Tests

Three commercial tests of EUROIMMUN AG (Lübeck, Germany) were included in this study. The EUROIMMUN *Echinococcus* ELISA is used for the detection of antibodies in alveolar and cystic echinococcosis patients and is based on *E. multilocularis* vesicle fluid (EmVF-ELISA). The EUROIMMUN Anti-*Echinococcus granulosus* IFAT (immunofluorescence antibody test) is used for the detection of antibodies against *E. granulosus s.l.* and *E. multilocularis*, containing frozen tissue sections of *E. granulosus s.s.* protoscolices (EgP-IFAT). Both tests are used for the screening of *Echinococcus* spp. infections in humans. For the species differentiation of *E. multilocularis* and *E. granulosus s.l.*, the Anti-Echinococcus-EUROLINE-Western blot was developed. This Western blot is designed for the confirmation of results from previously performed serological screening tests (EmVF-ELISA and EgP-IFAT) for the detection of *Echinococcus*-specific antibodies. This assay combines a whole native antigen extract (EmVF) and 3 specifically selected single recombinant antigens (EgAgB, Em18, and Em95). All serum samples that were evaluated in this study were randomized and anonymized and sent to EUROIMMUN for blind testing. The Western blot strips were analyzed by using the EUROLineScan software. The term ‘’Western blot-genus’’ was used if the test was able to diagnose both AE and CE. Conversely, the term ‘’Western blot-species’’ was used if the test provided correct species diagnosis (AE vs. CE).

The Vector-Best Echinococcus IgG ELISA (Vector-Best, Novosibirsk, Russia) was further included in this study. This diagnostic assay is the only test used for the routine diagnosis of both AE and CE in Kyrgyzstan. The assay was performed in our laboratory according to the manufacturer’s protocol.

#### 2.4.5. Enzyme-Linked Immunosorbent Assay (ELISA)

All ELISAs were performed in principle as described by Schweiger et al., (2012) [20] and the antigens were coated according to previous titration studies [20]. In this study, an anti-human-immunoglobulin G (IgG; polyclonal rabbit anti-Human-IgG, specific for the Fc part of the heavy chain; DAKO, Glostrup, Denmark) conjugated to alkaline phosphatase (Roche Applied Science, Rotkreuz, Switzerland) was used with a dilution of 1:500. Positive standard sera of human patients with confirmed AE or CE and negative standard sera of blood donors were included in all test runs and ELISA plates (5 sera per plate). 

### 2.5. Statistical Analysis & Evaluation of Test Performances 

Frequency distributions of ELISA absorbance values for patients with confirmed AE or CE or negative blood donors or ultrasound-negative individuals were assumed to follow a parametric distribution. Each set of absorbance values was fitted to normal, lognormal gamma and weibull distributions and the best fit was selected by the lowest AIC. The cut-off value of the absorbance for each sensitivity ranging in 100 steps between 0 and 1 was calculated from the area under the curve for the excess above that percentile. For example, if the density of the absorbance values representing the positive confirmed AE patients follows a normal distribution, the absorbance value representing a sensitivity of 90% will be the value above which 90% of that normal distribution lies. For each of the resulting 100 absorbance values, the specificity was estimated from the probability distribution of the absorbance from the blood donors or ultrasound negatives. Thus, if the absorbance value for the sensitivity of 90% was 0.15, then the specificity for that absorbance value was estimated as the area under the curve of the probability distribution with the upper bound of 0.15. This resulted in 100 pairs of sensitivity and specificity values. The absorbance value of the cut-off was determined as the value where the sum of the sensitivity and specificity values was maximum. For the test combinations it was assumed that at least one positive test indicated AE or CE. If neither test was positive, it indicated a negative result. The sensitivity of this parallel testing was estimated on the number of confirmed AE or CE patients that were positive on at least one of the two tests from the total number of AE or CE patients. Specificity was estimated based on the number of samples which were negative on both tests from either the healthy blood donors or the ultrasound-negative subjects. Exact binomial confidence intervals were calculated for the sensitivity of the parallel tests from the number of individuals defined as testing positive and the sample size of echinococcosis patients. Likewise, confidence intervals for the specificity were calculated from the numbers of individuals in the blood donor or ultrasound-negative group testing negative for both tests and the total numbers of individuals in these groups. For the evaluation of single test performances, we used McNemar’s test to compare all tests (Table 3 and Table 5). *P* values below 0.05 were considered as significant, *p* values above 0.05 were considered as not significant (N/S). If two assays are discriminating from each other with a *p* value below 0.05, the sensitivities of both assays can be compared to find the superior test with the higher sensitivity. All statistical analysis was undertaken in R (R Foundation for Statistical Computing, Vienna, Austria). 

### 2.6. Excluded Diagnostic Assays

We have excluded metacestode crude antigens derived from gerbils (*E. multilocularis*) and sheep (*E. granulosus s.s.*) due to high background reactions with the anti-human conjugate. The same issues were observed when using metacestode crude antigens for the on-plate purification with the monoclonal antibodies Em2G11 and EmG3. We have also excluded the data of the on-plate purification with mAb Em2G11, which gave the same results as mAb EmG3. Both mAbs are binding the native Em2 antigen [46].

## 3. Results

### 3.1. General Remarks

In this study we have evaluated and compared 17 diagnostic assays to detect specific serum antibodies against a variety of *Echinococcus* antigens in treatment-naïve AE and CE patients in Switzerland and Kyrgyzstan. We have calculated country-specific cut-off values based on 68 blood donors from Switzerland or 68 ultrasound-negative residents from Kyrgyzstan. Furthermore, we have included sera of 38 patients with non-parasitic liver lesions (NPLL) and 144 samples from various parasitic diseases to assess specificity and cross-reactivity. All assays were compared by McNemars’s test and optimal test-combinations for each country were calculated.

Data concerning all antigens and commercial tests that were analyzed in this study are summarized in Table 2. For the native antigens (EmVF, EmVC, EmP) and native purified antigens (Em2G11 and mAb EmG3-EmVC on-plate purification) we have evaluated one isolate from Switzerland and one from Kyrgyzstan, respectively. All antigens of both isolates did not differ significantly from each other by comparing them with McNemar’s test (data available in Supplementary Tables S1 and S2). Therefore, we have only included the results of the Swiss isolate of *E. multilocularis* for the evaluation of test performances. All exact values of sensitivities and specificities are provided in Supplementary Tables S1 and S2 with 95% confidence intervals (CIs) in brackets. Population-based specificities of blood donors in Switzerland and ultrasound-negative residents from Kyrgyzstan were not evaluated for commercial assays, due to a given cut-off value. Tests that are considered specific for *E. multilocularis* (Em2G11, recEm18 and recEm95) were excluded from the evaluation of assays for CE patients. Nevertheless, the cross-reactive potential of these tests for CE patients is shown in Table 3.

**Table 2:** Antigens and commercial tests for the serological diagnosis of alveolar and cystic echinococcosis used in this study.

ELISA */Commercial Test **	Origin	Remarks	Citation or Material & Methods
EmVF *^CH/KG^* *	*E. multilocularis*	in vitro vesicle fluid	Schweiger et al., 2011 [20]
EmVC *^CH/KG^* *	*E. multilocularis*	in vitro vesicle crude antigen	2.4.1 Vesicle crude antigens
EmP ^CH/KG^*	*E. multilocularis*	protoscolex crude antigen from *Meriones*	Schweiger et al., 2011 [20]
Em2G11 ^CH/KG^*	*E. multilocularis*	affinity-purified Em2G11 antigen from EmVC	Schweiger et al., 2011 [20]
mAb EmG3-EmVC ^CH/KG^*	*E. multilocularis*	on-plate purification with monoclonal antibody	2.4.2 On-plate purification
recEm18 *	recombinant	recombinant Em18 antigen	Schweiger et al., 2011 [20]
recEm95 *	recombinant	recombinant Em95 antigen	Gauci et al., 2002 [35]
EgVC *	*E. granulosus s.s.*	in vitro vesicle crude antigen	2.4.1 Vesicle crude antigens
mAb EmG3-EgVC *	*E. granulosus s.s.*	on-plate purification with monoclonal antibody	2.4.2 On-plate purification
EgCF *	*E. granulosus s.s.*	cyst fluid from sheep	Schweiger et al., 2011 [20]
EgP *	*E. granulosus s.s.*	protoscolex crude antigen from sheep	Schweiger et al., 2011 [20]
EgAgB *	*E. granulosus s.s.*	Antigen B from cyst fluid from sheep	Schweiger et al., 2011 [20]
recEg2B2 *	recombinant	recombinant 2B2 antigen	Hernández-González et al., 2012 [38]
Western blot-species **	EUROIMMUN AG	for species diagnosis (AE vs. CE)	2.4.4 Commercial Tests
Western blot-genus **	EUROIMMUN AG	for genus diagnosis (AE or CE)	2.4.4 Commercial Tests
EmVF-ELISA **	EUROIMMUN AG	*E. multilocularis* vesicle fluid ELISA	2.4.4 Commercial Tests
EgP-IFAT **	EUROIMMUN AG	*E. granulosus s.s.* protoscolices IFAT	2.4.4 Commercial Tests
VectorBest-ELISA **	VectorBest	Echinococcus-IgG-EIA-BEST	2.4.4 Commercial Tests

* All antigens were evaluated with ELISA by direct coating or by on-plate purification with monoclonal antibodies (mAb). ** Commercial tests: EUROIMMUN (EUROLINE-Western blot, EmVF-ELISA, EgP-IFAT), VectorBest (VectorBest-ELISA). ^CH/KG^ These antigens were produced with an Asian and a European isolate of E. multilocularis.

### 3.2. Alveolar Echinococcosis, Diagnostic Performance of Single Tests

The sensitivities and specificities of all tests for the serological diagnosis of AE in 60 Swiss patients and 40 Kyrgyz patients are summarized in Table 3. Population-based specificities were calculated with 68 blood donors from Switzerland and 68 ultrasound-negative residents from Kyrgyzstan. Furthermore, sera of patients with non-parasitic liver lesions, NPLL (N = 38) were included for Swiss AE patients to calculate the specificity for a clinical set-up. Cross-reaction rates with sera from 144 patients with non-*Echinococcus* parasitic infections and 64 CE patients were further determined. *P* values of McNemar’s test are also provided in Table 3. 

Switzerland: For the diagnosis of AE, most assays were equally sensitive and specific and did not discriminate significantly from each other. The best assays were native and native-purified antigens of *E. multilocularis* and *E. granulosus s.s.* (EmVF, EmVC, EmP, Em2G11, EmG3-EmVC on-plate purification, EgCF, EgP and EgAgB). Sensitivities in those tests ranged from 91 to 96%, and specificities ranged from 97 to 99% in blood donors and from 95 to 100% in the non-parasitic liver lesion (NPLL). Significantly less-sensitive tests were recEm95, EgVC and recEg2B2. Commercial tests had similar values compared to native antigens, with only VectorBest-ELISA and the EUROIMMUN Western blot for species diagnosis being less sensitive and specific as compared to other tests.

**Table 3:** Diagnostic performance of single assays for the diagnosis of alveolar echinococcosis (red for Switzerland, blue for Kyrgyzstan).

	Switzerland																							Kyrgyzstan			
**Number of sera**	**60**	**68**	**38**	**144**	**64**	McNemar’s Test: *p* values < 0.05 are significant (N/S = not significant)																		**40**	**68**	**144**	**64**
**Antigens & Methods**	**Sensitivity AE patients**	**Specificity blood donors**	**Specificity NPLL**	**Cross-reactions parasites**	**Cross-reactions CE**	EmVF-ELISA *	EmVC-ELISA *	EmP-ELISA *	Em2G11-ELISA *	EmG3-EmVC-ELISA *	**recEm18-ELISA**	**recEm95-ELISA**	**EmG3-EgVC-ELISA**	**EgVC-ELISA**	**EgCF-ELISA**	**EgP-ELISA**	**EgAgB-ELISA**	**recEg2B2-ELISA**	Western blot-genus **	Western blot-species **	EmVF-ELISA **	EgP-IFAT **	VectorBest-ELISA **	**Sensitivity AE patients**	**Specificity US negative**	**Cross-reactions parasites**	**Cross-reactions CE**
EmVF-ELISA *	0.96	0.99	0.95	0.19	0.86		N/S	N/S	**0.00**	N/S	N/S	**0.00**	N/S	N/S	N/S	N/S	N/S	N/S	N/S	N/S	N/S	N/S	N/S	0.74	0.89	0.11	0.68
EmVC-ELISA *	0.95	0.99	0.97	0.19	0.67	N/S		N/S	**0.02**	N/S	**0.03**	**0.02**	N/S	N/S	N/S	N/S	N/S	N/S	N/S	N/S	N/S	N/S	N/S	0.82	0.91	0.15	0.64
EmP-ELISA *	0.96	0.98	0.95	0.08	0.59	N/S	N/S		**0.02**	N/S	**0.03**	**0.02**	N/S	N/S	N/S	N/S	N/S	N/S	N/S	N/S	N/S	N/S	N/S	0.82	0.95	0.10	0.61
Em2G11-ELISA *	0.91	0.97	1.00	0.29	0.53	N/S	N/S	N/S		**0.01**	**0.00**	N/S	N/S	**0.01**	**0.02**	**0.02**	**0.01**	N/S	**0.02**	**0.00**	**0.02**	N/S	**0.03**	0.80	0.88	0.38	0.56
EmG3-EmVC-ELISA *	0.96	0.98	0.97	0.17	0.66	N/S	N/S	N/S	N/S		**0.02**	**0.05**	N/S	N/S	N/S	N/S	N/S	N/S	N/S	**0.03**	N/S	N/S	N/S	0.78	0.91	0.19	0.69
recEm18-ELISA	0.82	0.96	0.95	0.06	0.13	N/S	**0.01**	**0.02**	**0.03**	**0.02**		**0.00**	N/S	N/S	**0.03**	N/S	N/S	N/S	N/S	N/S	N/S	**0.02**	N/S	0.70	0.89	0.06	0.13
recEm95-ELISA	0.67	0.91	0.92	0.28	0.14	**0.00**	**0.00**	**0.00**	**0.00**	**0.00**	**0.00**		**0.01**	**0.01**	**0.01**	**0.00**	**0.01**	**0.01**	**0.02**	**0.00**	**0.03**	**0.05**	**0.01**	0.82	0.95	0.52	0.25
EmG3-EgVC-ELISA	0.78	0.83	0.86	0.22	0.66	N/S	N/S	N/S	N/S	N/S	N/S	**0.00**		N/S	N/S	N/S	N/S	N/S	N/S	**0.02**	N/S	N/S	N/S	0.78	0.83	0.22	0.66
EgVC-ELISA	0.75	0.83	0.92	0.19	0.53	**0.00**	**0.00**	**0.00**	**0.00**	**0.00**	N/S	N/S	**0.00**		N/S	N/S	N/S	N/S	N/S	**0.04**	N/S	N/S	**0.01**	0.73	0.67	0.22	0.41
EgCF-ELISA	0.95	0.99	0.97	0.38	0.86	N/S	N/S	N/S	N/S	N/S	N/S	**0.00**	N/S	**0.00**		N/S	N/S	N/S	N/S	**0.02**	N/S	N/S	N/S	0.76	0.88	0.29	0.73
EgP-ELISA	0.96	0.99	0.95	0.26	0.83	N/S	N/S	N/S	N/S	N/S	N/S	**0.00**	N/S	**0.00**	N/S		N/S	N/S	N/S	N/S	N/S	N/S	N/S	0.77	0.92	0.16	0.83
EgAgB-ELISA	0.89	0.98	0.95	0.35	0.83	N/S	**0.03**	N/S	N/S	N/S	N/S	**0.00**	N/S	N/S	N/S	N/S		N/S	N/S	**0.02**	N/S	N/S	N/S	0.70	0.81	0.40	0.84
recEg2B2-ELISA	0.64	0.64	0.84	0.41	0.58	**0.00**	**0.00**	**0.00**	**0.00**	**0.00**	**0.01**	N/S	**0.00**	N/S	**0.00**	**0.00**	**0.00**		N/S	**0.04**	N/S	N/S	N/S	0.65	0.65	0.40	0.84
Western blot-genus **	0.93	NA	0.97	0.10	0.83	N/S	N/S	N/S	N/S	N/S	**0.03**	**0.00**	N/S	**0.00**	N/S	N/S	N/S	**0.00**		**0.03**	N/S	N/S	N/S	0.65	NA	0.10	0.65
Western blot-species **	0.58	NA	0.97	0.11	0.56	**0.00**	**0.00**	**0.00**	**0.00**	**0.00**	**0.01**	N/S	**0.00**	N/S	**0.00**	**0.00**	**0.00**	N/S	**0.00**		N/S	**0.01**	N/S	0.45	NA	0.11	0.48
EmVF-ELISA **	0.88	NA	1.00	0.18	0.78	N/S	N/S	N/S	N/S	N/S	N/S	**0.00**	N/S	**0.04**	N/S	N/S	N/S	**0.01**	N/S	**0.01**		N/S	N/S	0.55	NA	0.18	0.52
EgP-IFAT **	0.90	NA	0.97	0.28	0.83	N/S	N/S	N/S	N/S	N/S	N/S	**0.00**	N/S	**0.00**	N/S	N/S	N/S	**0.01**	N/S	**0.01**	N/S		N/S	0.68	NA	0.28	0.74
VectorBest-ELISA **	0.77	NA	1.00	0.16	0.63	**0.01**	**0.00**	**0.00**	**0.00**	**0.00**	N/S	N/S	**0.01**	N/S	**0.00**	**0.00**	**0.02**	N/S	**0.00**	N/S	**0.02**	**0.01**		0.45	NA	0.16	0.35

* Antigens were produced in a duplicate with a Swiss and a Kyrgyz isolate and provided identical results. Presented here are the results from the Swiss isolate. ** Commercial tests: EUROIMMUN (EUROLINE-Western blot, EmVF-ELISA, EgP-IFAT), VectorBest (VectorBest-ELISA). NPLL: Non-parasitic liver lesions (e.g., liver cyst, neoplasia, cholangitis, etc.).

Kyrgyzstan: For the diagnosis of AE, the comparison of assays revealed similar results as for Switzerland, but with 10–20% lower values of sensitivities and specificities. However, the best assay for the diagnosis of AE in Kyrgyzstan was the recEm95-ELISA. This assay performed significantly better as compared to the other tests, except for Em2G11-ELISA. The sensitivity of recEm95 antigen was 82% with a specificity of 95% in the ultrasound-negative population, but with cross-reacting values of 52% in various parasitic diseases and 25% in CE patients. In comparison, the sensitivity for the Em2G11 antigen was 80% with a specificity of 88% in ultrasound-negative residents with 20% cross-reactions with other parasites and 56% with CE patients. Commercial tests were equally sensitive and specific when compared to the other tests, but not better than recEm95 and Em2G11.

#### 3.2.1. Subclinical Alveolar Echinococcosis, Diagnostic Performance of Single Tests 

For the subclinical diagnosis of 10 Swiss AE patients with small lesions (<2.5 cm), ELISAs using EmVC, Em2G11, and EmVF antigens revealed positive reactions in 10 out of 10 patients. This is followed by 9 out of 10 positive reactions in mAb EmG3-EmVC on-plate purification, EgP, EgCF, EgAgB, and recEg2B2; 8 out of 10 in EmP, recEm18, and mAb EmG3-EgVC (on-plate purification); 6 out of 10 in EgVC, and 3 out of 10 patients in recEm95. For the commercial tests, the EUROIMMUN Western blot, EmVF-ELISA, and EgP-IFAT were positive in 8 out of 10 patients, followed by the VectorBest-ELISA with 7 out of 10 patients.

#### 3.2.2. Alveolar Echinococcosis, Confirmation Tests

For a specific diagnosis of AE in Switzerland and Kyrgyzstan, we have evaluated all antigens derived from *E. multilocularis*. We have increased the cut-off values in our diagnostic tests to increase the specificity. All tests are summarized in Table 4. There was no difference in the sensitivity and specificity for AE patients from Kyrgyzstan and Switzerland by coating the EmVC antigen directly to the ELISA plate or by purifying the EmVC antigen with mAb EmG3 or mAb Em2G11 (on-plate purification of Em2 antigen). 

Switzerland: A sensitive and specific diagnosis of AE patients can be achieved with most antigens of *E. multilocularis* by increasing the cut-off value, except for recEm95. The sensitivity of recEm95 was 45% with a specificity in blood donors of 100%, 8% cross-reactions in various parasites, and 2% cross reactions with CE patients. In contrast, the sensitivity of all other antigens ranged from 70–90%, with a specificity in blood donors of 100%, 1–3% cross-reactions in patients infected with various parasites, and 12–34% cross reactions with CE patients. The crude antigens EmP and EmVF revealed the highest proportions of cross-reactions in CE patients with 51% and 66%, and EmVF had the highest cross-reactions in various parasitic diseases with 11%. The assays with the lowest cross-reactions in CE patients were recEm95 (2%) and recEm18 (12%) 

Kyrgyzstan: A specific diagnosis in AE patients can be achieved with most antigens of *E. multilocularis* by increasing the cut-off value, except for EmVF. The sensitivity of EmVF was 55%, with a specificity in blood donors of 91%, 11% cross-reactions in various parasites, and 66% cross reactions with CE patients. In contrast, the sensitivity of all other antigens ranged from 45–63% with a specificity in blood donors of 97–100%, 2–11% cross-reactions in patients infected with various parasites, and 0–52% cross reactions with CE patients (Table 4). The assays with the lowest cross-reactions in CE patients were recEm18 (0%) and recEm95 (9%).

**Table 4:** Evaluation of antigens in ELISA for the serological confirmation of alveolar echinococcosis.

	Sensitivity AE Patients	Specificity Blood Donors	Specificity NPLL Patients	Cross-Reactions Parasitic Infections	Cross-Reactions CE Patients
Number of Sera	60	68	38	144	64
Switzerland					
Antigens for ELISA					
EmVC *	0.87 (0.75–0.94)	1.00 (0.94–1.00)	0.97 (0.86–0.99)	0.01 (0.00–0.04)	0.20 (0.11–0.32)
mAb EmG3-EmVC *	0.87 (0.75–0.94)	1.00 (0.94–1.00)	0.97 (0.86–0.99)	0.02 (0.00–0.06)	0.34 (0.23–0.47)
Em2G11 *	0.77 (0.64–0.87)	1.00 (0.94–1.00)	1.00 (0.91–1.00)	0.03 (0.00–0.07)	0.17 (0.09–0.29)
mAb Em2G11-EmVC *	0.77 (0.64–0.87)	1.00 (0.94–1.00)	1.00 (0.91–1.00)	0.02 (0.00–0.06)	0.20 (0.11–0.32)
EmP *	0.90 (0.79–0.96)	1.00 (0.94–1.00)	0.97 (0.86–0.99)	0.03 (0.00–0.07)	0.51 (0.39–0.64)
EmVF *	0.87 (0.75–0.94)	1.00 (0.94–1.00)	0.97 (0.86–0.99)	0.11 (0.06–0.17)	0.66 (0.53–0.77)
recEm18	0.70 (0.57–0.81)	1.00 (0.94–1.00)	0.97 (0.86–0.99)	0.03 (0.00–0.07)	0.12 (0.06–0.23)
recEm95	0.45 (0.31–0.58)	1.00 (0.94–1.00)	0.95 (0.82–0.99)	0.08 (0.04–0.14)	0.02 (0.00–0.08)
Number of Sera	40	68	38	144	64
Kyrgyzstan					
Antigens for ELISA					
EmVC *	0.63 (0.46–0.77)	0.97 (0.90–0.99)	N/A	0.11 (0.06–0.17)	0.52 (0.39–0.64)
mAb EmG3-EmVC *	0.58 (0.41–0.73)	0.99 (0.92–0.99)	N/A	0.02 (0.00–0.06)	0.35 (0.23–0.47)
Em2G11 *	0.50 (0.34–0.66)	0.99 (0.92–0.99)	N/A	0.07 (0.03–0.12)	0.17 (0.09–0.29)
mAb Em2G11-EmVC *	0.50 (0.34–0.66)	0.99 (0.92–0.99)	N/A	0.02 (0.00–0.06)	0.22 (0.15–0.40)
EmP *	0.63 (0.46–0.77)	0.99 (0.92–0.99)	N/A	0.05 (0.02–0.10)	0.52 (0.39–0.64)
EmVF *	0.55 (0.38–0.71)	0.91 (0.82–0.97)	N/A	0.11 (0.06–0.17)	0.66 (0.53–0.77)
recEm18	0.45 (0.29–0.62)	0.99 (0.92–0.99)	N/A	0.03 (0.00–0.07)	0.00 (0.00–0.06)
recEm95	0.58 (0.41–0.73)	1.00 (0.94–1.00)	N/A	0.06 (0.03–0.12)	0.09 (0.04–0.19)

* All four assays are targeting the native antigen Em2 by direct coating of the affinity-purified antigen or by on-plate purification with mAbs.

### 3.3. Cystic echinococcosis, Diagnostic Performance of Single Tests

The diagnostic sensitivities and specificities for CE patients in Switzerland (N = 41) and Kyrgyzstan (N = 23) are summarized in Table 5. Population-based specificities were calculated based on 68 blood donors from Switzerland and 68 ultrasound-negative residents from Kyrgyzstan. Furthermore, sera of patients with non-parasitic liver lesions, NPLL (N = 38) were included for Swiss CE patients to calculate the specificity for a clinical set-up. Cross-reaction rates with sera from 144 patients with non-*Echinococcus* parasitic infections and 100 AE patients were further determined. *P*-values of McNemar’s test are also provided in Table 5. 

Switzerland: For the diagnosis of CE, most assays were equally sensitive and specific and did not significantly differ from each other. The best assays were native crude and native purified antigens of *E. multilocularis* and *E. granulosus s.s.* (e.g., EmVF, EmVC, EmP, EmG3-EmVC on-plate purification, EgCF, EgP and EgAgB). Diagnostic sensitivities for those tests ranged from 81 to 94% and specificities from 89 to 99% in blood donors and from 92 to 100% regarding non-parasitic liver lesions (NPLL). A significantly lower sensitivity was found for EgVC and recEg2B2. Commercial tests had similar values as native antigens, only VectorBest-ELISA and the EUROIMMUN Western blot for species diagnosis were less sensitive and specific as compared to other tests.

**Table 5:** Diagnostic performance of single assays for the diagnosis of cystic echinococcosis (red for Switzerland, blue for Kyrgyzstan).

	Switzerland																				Kyrgyzstan			
**Number of Sera**	**41**	**68**	**38**	**144**	**100**	McNemar’s Test: *p* values < 0.05 are significant (N/S = not significant)															**23**	**68**	**144**	**100**
**Antigens & Methods**	**Sensitivity CE patients**	**Specificity blood donors**	**Specificity NPLL**	**Cross-reactions parasites**	**Cross-reactions AE**	EmVF-ELISA *	EmVC-ELISA *	EmP-ELISA *	EmG3-EmVC-ELISA *	**EmG3-EgVC-ELISA**	**EgVC-ELISA**	**EgCF-ELISA**	**EgP-ELISA**	**EgAgB-ELISA**	**recEg2B2-ELISA**	Western blot-genus **	Western blot-species **	EmVF-ELISA **	EgP-IFAT **	VectorBest-ELISA **	**Sensitivity CE patients**	**Specificity US negative**	**Cross-reactions parasites**	**Cross-reactions AE**
EmVF-ELISA *	0.94	0.97	0.95	0.34	0.87		N/S	N/S	N/S	N/S	N/S	N/S	N/S	N/S	**0.00**	N/S	**0.03**	N/S	N/S	N/S	0.70	0.85	0.14	0.76
EmVC-ELISA *	0.81	0.89	0.95	0.35	0.92	N/S		N/S	N/S	N/S	N/S	N/S	N/S	N/S	**0.00**	N/S	N/S	N/S	N/S	N/S	0.66	0.86	0.28	0.90
EmP-ELISA *	0.84	0.96	0.92	0.26	0.83	N/S	N/S		N/S	N/S	N/S	N/S	N/S	N/S	**0.00**	N/S	N/S	N/S	N/S	N/S	0.76	0.94	0.26	0.83
EmG3-EmVC-ELISA *	0.84	0.93	0.97	0.23	0.88	N/S	N/S	N/S		N/S	N/S	N/S	N/S	N/S	**0.01**	N/S	N/S	N/S	N/S	N/S	0.67	0.88	0.29	0.86
EmG3-EgVC-ELISA	0.82	0.90	0.82	0.31	0.85	**0.00**	**0.04**	N/S	**0.03**		**0.02**	**0.03**	N/S	N/S	**0.00**	N/S	**0.02**	**0.03**	N/S	N/S	0.67	0.78	0.27	0.83
EgVC-ELISA	0.61	0.76	0.84	0.33	0.79	**0.00**	**0.01**	**0.01**	**0.00**	N/S		N/S	**0.04**	N/S	N/S	N/S	N/S	N/S	N/S	N/S	0.42	0.80	0.12	0.57
EgCF-ELISA	0.93	0.97	0.97	0.43	0.91	N/S	N/S	N/S	N/S	N/S	**0.00**		N/S	N/S	**0.02**	N/S	N/S	N/S	N/S	N/S	0.68	0.88	0.29	0.81
EgP-ELISA	0.93	0.97	0.92	0.32	0.86	N/S	N/S	N/S	N/S	**0.04**	**0.00**	N/S		N/S	**0.00**	N/S	**0.02**	**0.03**	N/S	N/S	0.76	0.93	0.17	0.78
EgAgB-ELISA	0.86	0.96	0.92	0.39	0.79	N/S	N/S	N/S	N/S	**0.04**	**0.00**	N/S	N/S		**0.02**	N/S	N/S	N/S	N/S	N/S	0.68	0.92	0.34	0.73
recEg2B2-ELISA	0.49	0.84	0.92	0.08	0.33	**0.00**	**0.00**	**0.00**	**0.00**	**0.00**	N/S	**0.00**	**0.00**	**0.00**		**0.01**	N/S	N/S	**0.01**	N/S	0.45	0.86	0.08	0.33
Western blot-genus **	0.83	N/A	0.97	0.10	0.93	N/S	N/S	N/S	N/S	N/S	**0.00**	N/S	N/S	N/S	**0.00**		N/S	N/S	N/S	N/S	0.65	N/A	0.10	0.65
Western blot-species **	0.56	N/A	0.97	0.11	0.58	**0.00**	**0.00**	**0.00**	**0.00**	**0.03**	N/S	**0.00**	**0.00**	**0.00**	N/S	**0.00**		N/S	N/S	N/S	0.48	N/A	0.11	0.45
EmVF-ELISA **	0.78	N/A	1.00	0.18	0.88	N/S	N/S	N/S	N/S	N/S	**0.01**	N/S	N/S	N/S	**0.00**	N/S	**0.01**		N/S	N/S	0.52	N/A	0.18	0.55
EgP-IFAT **	0.83	N/A	0.97	0.28	0.90	N/S	N/S	N/S	N/S	N/S	**0.00**	N/S	N/S	N/S	**0.00**	N/S	**0.00**	N/S		N/S	0.74	N/A	0.28	0.68
VectorBest-ELISA **	0.63	N/A	1.00	0.16	0.77	**0.02**	**0.02**	N/S	**0.03**	N/S	N/S	**0.03**	**0.02**	**0.02**	**0.02**	N/S	N/S	N/S	**0.03**		0.35	N/A	0.16	0.45

* Antigens were produced in duplicate with a Swiss and a Kyrgyz isolate and provided identical results. Presented here are the results from the Swiss isolate. ** Commercial tests: EUROIMMUN (EUROLINE-Western blot, EmVF-ELISA, EgP-IFAT), VectorBest (VectorBest-ELISA). NPLL: Non-parasitic liver lesions (e.g., liver cyst, neoplasia, cholangitis etc.).

Kyrgyzstan: For the diagnosis of CE, the comparison of assays yielded similar results as those found in Swiss patients, but with 10–20% lower sensitivities and specificities. The sensitivities for most antigens ranged from 66 to 76% and specificities ranged from 80 to 94% in ultrasound-negative residents. The antigen recEg2B2 was less sensitive and specific compared to 10 out of 15 assays. Commercial tests were equally sensitive and specific as compared to most of the other tests. In general, no assay showed a high sensitivity combined with low cross-reactions in patients with various parasitic diseases nor with AE patients.

### 3.4. Cross-Reactions of Patients with Non-Echinococcus Parasitic Infections for the Serological Diagnosis of Alveolar Echinococcosis

The proportion of cross-reactions determined with 144 sera of patients with non-*Echinococcus* parasitic infections for the serological diagnosis of AE in Switzerland and Kyrgyzstan are summarized in Table 6. In this analysis we have used country-specific cut-off values, resulting in country-specific results. The proportions of cross-reactions were similar with the cut-offs for Switzerland and Kyrgyzstan. The lowest cross-reactivity of 6.3–8.3% was found for the EmP antigen, 5.6% for the recEm18 antigen, and 9.7% and 11.1% for the EUROIMMUN Western blot-genus and species diagnosis, respectively. All other commercial tests had similar values compared to assays derived from native or recombinant antigens. Most native antigens showed various cross-reactions in 10–40% of serum samples from 144 various parasitic infections.

### 3.5. Cross-Reactions of Patients with non-Echinococcus Parasitic Infections for the Serological Diagnosis of Cystic Echinococcosis

The proportion of cross-reactions determined with 144 sera of patients with non-*Echinococcus* parasitic infections for the serological diagnosis of CE in Switzerland and Kyrgyzstan are summarized in Table 7. In this analysis we have used country-specific cut-off values, resulting in country-specific results. The proportions of cross-reactions were similar to the cut-off values for Switzerland and Kyrgyzstan. The lowest cross-reactivity of 8.3% was found for the recEg2B2 antigen and 9.7% for the EUROIMMUN Western blot-genus diagnosis. All other commercial tests had similar values compared to assays derived from native or recombinant antigens. Most native antigens showed various cross-reactions in 20–40% of serum samples from 144 various parasitic infections.

### 3.6. Diagnostic Performance of Test Combinations for the Serological Diagnosis of Alveolar Echinococcosis

The sensitivities and specificities of two-test-combinations (ELISA) for the serological diagnosis of AE in Switzerland and Kyrgyzstan are summarized in Table 8. The best antigen combination for the diagnosis of AE in Switzerland was the use of EmVF and EgCF antigens with a sensitivity and specificity both of 100%. Other test combinations reached similar values, like EmVC and EgCF (Se: 97%; Sp: 100%), EmVC and Em2G11, or EmVC and EgP (Se: 97%; Sp: 99%). The best antigens for the diagnosis of AE in Kyrgyzstan were EmP and recEm95 with a sensitivity of 98% and a specificity of 87%. The test combination of Em2G11 and recEm95 in Kyrgyzstan had similar values with a sensitivity of 98% and a specificity of 81%. Equivalent sensitivities and specificities could be achieved by combining the Em95 antigen with most native antigens of *E. multilocularis* and *E. granulosus s.s*.

### 3.7. Diagnostic Performance of Test Combinations for the Serological Diagnosis of Cystic Echinococcosis

The sensitivities and specificities of two-test-combinations (ELISA) for the serological diagnosis of CE in Switzerland and Kyrgyzstan are summarized in Table 9. The best test combination for the clinical diagnosis of CE in Switzerland was EgP and EgCF with a sensitivity of 90% and a specificity of 100%. By combining EgAgB with either EgP (Se: 93%; Sp: 99%) or EgAgB with EgCF (Se: 90%; Sp: 99%), similar values could be achieved. The best assay for the diagnosis of CE in Kyrgyzstan was EgP and recEg2B2 with a sensitivity of 87% and a specificity of 89%. 

## 4. Discussion

Over the recent decades, a vast number of diagnostic laboratory assays have been developed, focusing on specific serum antibody detection in echinococcosis patients. Therefore, we have included newly developed assays and the most acquainted antigens used in ELISA to evaluate their diagnostic performance. The most frequently used antigens presently are derived from native metacestode tissue from intermediate hosts or from parasite larval stages grown in vitro. Native metacestode antigens usually show a high sensitivity but are tagged by cross-reactions within *Echinococcus* species and other parasites [18,20]. 

Our results indicate that the use of native antigens from *Echinococcus* species are still some of the best antigens for serology; however, they have limitations in view of cross-reactivity and show disparities in lot-to-lot reproducibility to some extent. For commercial tests as well as for laboratory-made tests, a careful validation of each antigen lot is required. Regarding the sensitivity, specificity, and cross-reactions in our evaluation, the use of EgCF, EgP, EmVF, EmP, and EmVC did not show significant disparities from each other in both evaluated countries (Table 3 and Table 5). For the direct ELISAs we have evaluated native antigens from sheep (EgCF, EgP, and AgB) and *Meriones* (EmP). Compared to in vitro-derived antigens of *Echinococcus* spp., these native antigens showed likewise relatively high cross-reactions with antibodies from patients with various parasitic diseases (17–38%) and similar diagnostic sensitivities. The use of in vitro-derived antigens did statistically not yield better test performances compared to antigens from intermediate hosts. Only the antigen EmP showed lower cross-reactions (6–8%) with 144 serum samples from patients with other parasitic infections. One reason for this might be that this antigen was produced by affinity purification of *E. multilocularis* protoscolices using the monoclonal antibody Em2G11. With this additional purification step, small vesicles and parasite particles can be removed from the sieved protoscolices by arguably eliminating cross-reactive epitopes from the metacestode mixture [20]. Similar reasons could hold true for the high cross-reactivity of 29–38% with various parasitic diseases regarding the EgP antigen. The *Echinococcus granulosus s.l.* purified protoscolices fraction still contains tissue particles from both the laminated and the germinal layer, including microvesicles surrounded by a laminated layer. In countries endemic for various parasitic (especially helminthic) diseases, potential cross-reactivity may become a major factor in the sero-diagnosis of AE. Thus, the consideration of EmP antigen could be of use to reduce cross-reactions in diagnostic assays by maintaining a high sensitivity.

For the production of native antigens from *E. multilocularis*, we have evaluated two geographically and genetically different isolates, one from Switzerland and one from Kyrgyzstan, (2.3 parasite isolates). No differences were found based on the origin of the two isolates by producing the native antigens EmVF, EmVC, EmP, Em2G11, and mAb EmG3-EmVC on-plate purification with the two isolates. Thus, we have only included the European isolate of *E. multilocularis* for the native and in vitro antigens in the results section (EmVF, EmVC, EmP, Em2G11, and mAb EmG3-EmVC on-plate purification). The European isolate of *E. multilocularis* did not show significant differences from antigens derived from the Asian isolate in AE patients from both endemic areas. The values obtained for sensitivity and specificity were differing only within a few percent amongst all antigens deriving from the two isolates (Supplementary Table S1). So far no comprehensive study has compared the diagnostic properties of two different haplotypes of *E. multilocularis*. Our results indicate that a global use of *E. multilocularis* antigens seems to be suitable, regardless of the origin of the isolate. 

We could not observe apparent differences in the cut-off values of assays in AE patients from Switzerland or Kyrgyzstan. Hence, the differences in test performances are more likely linked to the host reaction, i.e., two different patient populations in Switzerland and Kyrgyzstan, and not to the origin of the antigens or the isolates. Similar replicability in *Echinococcus* spp. native antigens for ELISA could be observed in other studies where there was no statistical difference in antigen batches or preparations, regardless of some variability in cut-off values [20,53]. 

Historically, the problems regarding cross-reactivity have been experimentally addressed for a long time already, e.g., by developing a native purified carbohydrate antigen named Em2 for the diagnosis of AE. The diagnostic potential of the Em2 antigen was later confirmed for the specific diagnosis of AE patients with few cross-reactions of 5–20% in CE patients and 0–5% in other parasites [21,24,25,29]. In the Swiss part of our study, the antigen Em2G11 was cross-reacting in 53% of the CE patients and 29% of other parasitic diseases. In Kyrgyzstan, similar values could be observed in 56% of CE patients and 20% of other parasitic diseases (Table 3). Nevertheless, the specificity of Em2G11 could be improved by increasing the cut-off values as shown in Table 4. No statistical difference was observed when purifying the Em2G11 antigen from EmVC with the mAb EmG3 or mAb Em2G11 via on-plate purification. Interestingly, the same findings were observed when coating the *E. multilocularis* vesicle crude antigen (EmVC) directly to the ELISA plate. Both EmVC and Em2G11 can be used for a specific diagnosis of AE, after adapting the cut-off value to reach optimal specificities. Therefore, we suggest that the laborious production of Em2 and Em2G11 antigen via affinity purification can be replaced by the use of EmVC antigen directly. The EmVC antigen can be produced from metacestodes of *E. multilocularis*, by cultivating respective vesicles in vitro for a few months [49,50]. By using one heavily (intraperitoneally) infected rodent, dozens of cell-culture flasks can be initiated. We estimate that we can produce 50 mg of antigen per flask after 4–6 months of in vitro cultivation. This corresponds to 1000 ELISA plates or 96,000 ELISA single wells.

Furthermore, native metacestode crude antigens are often contaminated with host proteins (e.g., immunoglobulins, albumin and other host molecules) which have the potential to increase background reactions in diagnostic assays. These issues became evident in our analysis by using native *Echinococcus* spp. antigens derived from rodents or sheep (data not shown). Similar test performances were noted by Schweiger and colleagues (2012), where a metacestode crude antigen from gerbils had the lowest values of specificity compared to all other antigens [20].

Another method for the affinity purification of antigens is the on-plate purification by using monoclonal antibodies (mAb) in ELISA (2.4.2 On-plate purification). In our study we have selected mAb EmG3, which can be purified more easily compared to mAb Em2G11. Both mAbs are binding the same antigen Em2 or Em2G11. The use of native antigens from intermediate hosts was not suitable for the on-plate purification with monoclonal antibodies, due to an increase in unspecific binding (data not shown). A sensitive and specific on-plate purification assay was only adequate when using in vitro-derived antigens without host tissue. Therefore, we have evaluated solely in vitro-derived EmVC and EgVC antigens for the on-plate purification with mAb EmG3. However, the on-plate purification did not show statistically superior test performances compared to directly coated EmVC and EgVC antigens in ELISA, nor to affinity-purified Em2G11 antigen. We can argue that this additional purification step cannot eliminate cross-reacting epitopes from the antigens, nor increase the sensitivity and therefore the use of EmVC directly coated in ELISA is the most suitable assay.

By comparing the clinical data of patients from Switzerland and Kyrgyzstan there was one major difference that was of note (Table 1). One obvious disparity is the average age in Kyrgyz AE patients (males = 32, females = 28), compared to Swiss AE patients (males = 58, females = 54). This difference could be caused by a differing age pattern of the healthy population in Switzerland and Kyrgyzstan. The average age in Switzerland in 2020 was 43 years, compared to 26 years in Kyrgyzstan (www.statista.com, accessed on 23 March 2022). Therefore, also the age at infection in AE patients seems different in both countries by assuming the infection has happened years ago. Somehow the incubation period in Kyrgyzstan seems shorter than in Europe (usually 10–15 years). Frequent infections in kids and young adults in Kyrgyzstan have recently become evident with 26% of surgical AE patients below 19 years old and 26% of patients between 20–29 years old (N = 598) [15]. 

The clinical data regarding staging and lesion size of AE patients in Switzerland and Kyrgyzstan showed certain disparities from each other as well. There was a tendency for more P1N0M0 stages and small lesions in Switzerland, in contrast to slightly more progressive and larger lesions in Kyrgyzstan. However, due to partially missing data from Kyrgyzstan and potential varieties in diagnostic approaches and disease surveillance, these differences are delicate to compare. For CE patients, no noticeable difference in clinical data or age patterns between Kyrgyzstan and Switzerland was observed. In this context it is important to mention that only 2 out of 41 CE patients that were diagnosed in Switzerland were actually of Swiss origin. All other patients originated from countries southward of Switzerland.

In the Kyrgyzstan part of this study, all antigens showed overall 10–20% lower diagnostic values regarding sensitivity and specificity due to cross-reacting antibodies in the ultrasound negative population. The reason for this could be multifactorial. Our hypothesis is that the background in diagnostic tests is higher in Kyrgyzstan due to the relatively frequent occurrence of other parasitic diseases, especially in children. A recent study in Kyrgyzstan in Osh oblast with 1262 schoolchildren aged 6–15 years indicated that 41% of the kids harbored at least one helminth species. The most prevalent helminths were *Ascaris lumbricoides* (23.1%), *Enterobius vermicularis* (19.3%), followed by *Hymenolepis nana* (4.4%), *Fasciola hepatica* (1.9%), and *Dicrocoelium dendriticum* (1.8%) [54]. Our hypothesis is supported by the fact that most of our assays cross-react with other parasitic diseases (Table 6 and Table 7). Infections with *Ascaris lumbricoides* in particular interfere with all of our tests in 12.5–62.5% of serum samples. Similar values can be observed in infections with *F. hepatica*, *Strongyloides stercoralis*, and *Trichinella* spp. The highest cross-reacting values were seen in filarial infections, ranging between 12.5–93.8% for native antigens. One reason for the higher background in the Kyrgyz ultrasound-negative population might be that during adolescence, the infection pressure of helminths is presumably getting lower. However, after recurrent contact with helminths in endemic areas like Kyrgyzstan, the antibody response and low persisting antibody levels could interfere in the specific diagnosis of echinococcosis. This is supported by the fact that most of our antigens needed a higher cut-off value in the Kyrgyz population, and therefore we could not discriminate infected echinococcosis patients from uninfected persons with a high certainty as compared to Switzerland. Interestingly, both EmP and recombinant Em95 antigen had a relatively high sensitivity for the diagnosis of clinical AE patients in Kyrgyzstan (82%), with a specificity of 95% in the ultrasound-negative population. The recombinant Em95 antigen is derived from genes of *E. multilocularis*, similarly as described for Eg95 antigen, which is a penetration gland protein in oncospheres of *E. granulosus s.l.* [35]. We cannot find a reasonable explanation yet as to why the antibody response in Kyrgyzstan against recEm95 and EmP is elevated and seems to be even increasing in progressive AE patients for recEm95 (data not shown). Reasons therefore could be multifactorial, like changes in the gene expression in *E. multilocularis*. This could be related to immunological and genetic differences in populations of different geographical and ethnic origins, and also genetic differences in the local parasite strains/isolates. In particular, the Asian haplotype of *E. multilocularis* is of interest in Kyrgyzstan. This variant is responsible for all human AE patients [45]. Furthermore, recurrent contact with oncospheres or changes in the immunological response in Kyrgyz AE patients could play a role. However, the antibody response in the ultrasound-negative Kyrgyz population against recEm95 seems to be low (95% specificity). Therefore, after recurrent contact with oncospheres, also the uninfected population should have an increase in antibody levels against recEm95. 

A recent study in Kyrgyzstan reported low sensitivities of only 50–60% by analyzing 106 suspected AE patients in an ultrasound study with EgCF, EgP, EgAgB, Em2G11, and recEm18 antigens [14]. In our study we have included sera of 68 ultrasound-negative residents from the same study to calculate country-specific cut-off values. In comparison to the study conducted by Bebezov and colleagues, our study was not focusing on the subclinical diagnosis of AE in epidemiological population screenings with ultrasound, but rather on clinical AE patients that were seeking hospital treatment. Therefore, we can argue that a disease progression leads to an increase in antibody levels and hence higher values of sensitivities in diagnostic tests compared to the mentioned study. Already Bebezov and colleagues have found that lesions in AE patients below 2 cm are often serologically negative [14]. Unfortunately, we did not have a sufficient number of sera of confirmed subclinical AE patients from Kyrgyzstan to follow up on these findings. Nevertheless, we have analyzed sera of 10 confirmed AE patients from Switzerland with arguably rather early and subclinical infections (lesion sizes below 2.5 cm). In Switzerland, the subclinical diagnosis of AE seems not to be an issue by diagnosing all 10 patients with EmVC, Em2G11 or EmVF antigen. At least 9 out of 10 subclinical AE patients could be diagnosed with EgP, EgCF, EgAgB, recEg2B2, and mAb EmG3-EmVC on-plate purification. It is important to mention, that we have only few data about the lesion activity in those 10 confirmed patients, i.e., we do not know if the lesions were abortive. The serological response to recEm18 antigen was positive in 8 out of 10 patients, which rather indicates activity. In a recent study by Gottstein and colleagues, 10 AE patients that were considered abortive were all negative in recEm18 ELISA and therefore most likely inactive, but all of these patients were sero-positive in the EmVF Western blot, which is part of the EUROLINE-Western blot [28,29].

In general, we have not found a specific test for the diagnosis of CE neither in Kyrgyzstan nor in Switzerland. All of our native antigens derived from *E. granulosus s.l.* had an arguably high sensitivity of 86–93% for the diagnosis of CE in Switzerland, with a specificity in blood donors of 92–97%, 34–43% cross-reactions with various parasitic diseases, and 79–91% cross-reactions with AE patients. The sensitivities and specificities of the same antigens showed unequal results with 10–20% lower values for the diagnosis of CE in Kyrgyzstan, similarly to AE patients. Similar low test sensitivities of < 80% and specificities of < 90% as we have found in our study are known from the literature. In this regard, the antibody detection in CE patients from Kenia (Turkana) seems lower than in other parts of the world with sensitivities of native antigens in ELISA of only 50–60% [55]. In another study, the specificity for healthy people in Iran with EgCF-ELISA was 77%, with a sensitivity of 91% for CE patients [56]. We have found similar values for CE patients in Kyrgyzstan for the EgCF antigen of 68% sensitivity and 88% specificity. For the diagnosis of CE patients in Switzerland, the antigen EgCF shows values of 93% sensitivity and 97% specificity in our study, which could be observed earlier in a study in Switzerland with equal values [20]. The reasons therefore are most likely the difference in the negative population to evaluate the cut-off values in different countries. In Kyrgyzstan, the negative population for the evaluation of cut-off values was ultrasound negative for echinococcosis. Nevertheless, other parasitic infections and cross-reactions in a variety of serological assays cannot be excluded and are to be expected to some extent as described before. 

Some progress for the diagnosis of CE has been reported over the last years with the development of recombinant antigens derived from genes of Antigen B like recombinant antigen recEg2B2 [38]. A recent study with CE patients from Peru has reported a high sensitivity of the recEg2B2 antigen of 87.6% in 186 confirmed CE patients and a specificity of 99.1% in 110 blood donors. Widely used EgCF antigen showed a sensitivity of 83.3% in the confirmed CE patients and a specificity of 95.4% in the blood donors. Moreover, the number of cross-reactions in recEg2B2 antigen was lower (19.5%) in 174 samples, compared to 51.7% in EgCF [38]. In our study, the recEg2B2 antigen showed an arguably low sensitivity of 49% for the diagnosis of CE in Switzerland and 45% in Kyrgyzstan as compared to the results of the native antigens. Similar results were observed by comparing the specificity of recEg2B2 to native antigens. The specificity in blood donors in Switzerland was 13% lower in recEg2B2 as compared to native antigens of *E. granulosus s.l.*, like EgCF and EgP. 

To increase sensitivity and specificity in our assays, we have evaluated two-test combinations for the diagnosis of echinococcosis in Switzerland and Kyrgyzstan (Table 8 and 9). In Switzerland, most test combinations with native or recombinant antigens revealed an increase of the sensitivity and specificity up to both 100% in EgCF x EmVF for the diagnosis of AE. Numerous antigens had similar values over 96% sensitivity and specificity by combining native antigens of *Echinococcus* species. Hence, the strategy for the diagnosis of AE and CE should be critically evaluated by taking into consideration the cross-reactive potential of native antigens with various parasitic diseases and especially within *Echinococcus* species. In Switzerland, the infection with other parasitic diseases is expected to be low, but it should be taken into account that parasitic infections can persist after traveling in endemic countries or echinococcosis has to be excluded from the differential diagnosis list, e.g., due to eosinophilia or unclear liver lesions. Therefore it is of significant importance to combine diagnostic imaging with positive serological tests [6]. Another point to consider in countries with various parasitic diseases would be to combine specific antigens like recEm18 or EmP with other sensitive but rather unspecific native antigens to overcome the issues of heavy cross-reactions in single tests. In Kyrgyzstan, a combination of recombinant recEm95 antigen with EmP antigen for the diagnosis of AE seems to be suitable, with a combined sensitivity of 98% and a specificity of 87% in the ultrasound–negative population. Nevertheless, both antigens have the potential to cross-react with sera of CE patients; therefore, diagnostic imaging is crucial to support the serological diagnosis.

Furthermore, we have evaluated four commercial tests for the diagnosis of AE and CE from EUROIMMUN AG and VectroBest. In our comparison of tests, the EUROLINE-Western blot and EmVF-ELISA or EgP-IFAT were not significantly better than most of the native antigens tested by ELISA. By using the EUROLINE-Western blot in our study, a species diagnosis was achieved in 58% of AE patients and in 56% of CE patients from Switzerland. Higher values could be achieved by genus diagnosis in 93% of AE patients and 83% of CE patients in Switzerland. However, the EUROLINE-Western blot seems not to work well in Kyrgyzstan with the set cut-off values. A species diagnosis was achieved in 45% of AE patients and in 48% of CE patients in Kyrgyzstan. By genus diagnosis, both 65% of Kyrgyz AE and 65% of CE patients were positive. Those results are in agreement with a study by Deininger and Wellinghausen, 2019. They compared the Anti-Echinococcus-EUROLINE Western blot (ELB) to the LD-BIO™ Western blot (LDBio). Both Western blots showed similar findings for the species diagnosis of 66.7% (ELB) and 62.5% (LDBio), respectively. A positive test result for negative patients was reported in 8.3% (ELB) and in 4.2% (LDBio), but with unclear statistical significance [42]. Nevertheless, the EUROLINE-Western blot can be used in an automated reading system and can therefore be used in laboratories without access to native antigens of *Echinococcus* species. The use of recEm95 in ELISA and in the EUROLINE-Western blot showed some disparities from each other. These differences could be caused by the use of different antigen sequences and expression systems, antigen presentation upon test procedure, incubation techniques, and different reagents and read-outs. 

Moreover, the exclusive use of VectorBest-ELISA in Kyrgyzstan should be critically reconsidered, due to an overall significantly low sensitivity of 45% for AE and 35% for CE patients. Besides, the diagnostic performance of all commercial tests should be validated in different endemic areas to calculate country-specific cut-off values.

Our study showed some limitations for the serological diagnosis of echinococcosis patients. We were focusing on detecting serum antibodies in echinococcosis patients with a rather progressive form of the disease and who were diagnosed in hospitals. Therefore, our assays and clinical cut-off values have to be critically evaluated for screening programs in endemic areas where many patients may still remain asymptomatic or in an early stage of the disease, and such investigations should be combined with ultrasound. Furthermore, all assays should be evaluated with patients and control samples from countries where these assays are used, to evaluate country-specific cut-off values. Our data showed that our assays perform differently in two distinct countries and thus also in different patient groups, and therefore a local validation of assays is essential. 

## 5. Conclusions and Practical Recommendations

For the serological diagnosis of AE in Switzerland by ELISA, a panel of native antigens performed as sensitive and specific as compared to the recombinant antigen Em18 and most of the commercial tests. For a screening test by ELISA, we recommend using the EmP antigen, which had statistically lower cross-reactions with serum samples of various parasitic infections. The antigen EmVC can be used both for the confirmation and screening of AE patients in Switzerland. Overall, complementation of any ELISA-based serodiagnostic approach with Western blotting is recommended to yield optimal diagnostic performances in specialised laboratories with access to such a technology. 

For the diagnosis of AE in Kyrgyzstan by ELISA, the recEm95 antigen showed the best diagnostic values. However, a two-test combination of Em95 and EmP antigen achieved an optimal sensitivity of 98% with a specificity of 87%. This two-test combination should be further evaluated as a serological screening strategy in Kyrgyzstan or other endemic areas for an early disease surveillance in combination with ultrasound. 

For a sensitive diagnosis of CE in Switzerland and Kyrgyzstan, native antigens of *E. granulosus s.l.* like EgCF, EgP and Antigen B are still the best assays available. In our view, there is an urgent need to improve the specific diagnosis of CE. Given that antibodies against *E. granulosus s.l.* are heavily cross-reacting with other helminths, including *E. multilocularis*, a focus could be laid on a specific search for circulating parasite antigens or parasite excretory/secretory products like RNA and DNA. Furthermore, targeted proteomics are desirable to improve not only the diagnosis of *E. granulosus s.l.*, but for *E. multilocularis* as well. 

Conclusively, diagnostic assays including commercial tests should be evaluated locally with patients and control samples from the same region to calculate country-specific cut-off values. The claim of standardization of diagnostic assays [19] is in our view not feasible, due to variations in cut-off values and test performances in different endemic areas as shown in this study for Europe and Central Asia.

## Figures and Tables

**Table 6:** Assays for the serological diagnosis of alveolar echinococcosis—cross-reactions with 144 sera of patients with non-*Echinococcus* parasitic infections (Switzerland/CH, Kyrgyzstan/KG).

Parasitic Diseases (N = 144)	*Entamoeba*	*histolytica*	*Taenia*	*solium*	*Ascaris*	*lumbricoides*	*Trichinella*	spp.	*Strongyloides*	*stercoralis*	*Toxocara*	spp.	*Shistosoma*	spp.	*Fasciola*	*hepatica*	Filarial	Species	Total	Total
**Country**	CH	KG	CH	KG	CH	KG	CH	KG	CH	KG	CH	KG	CH	KG	CH	KG	CH	KG	CH	KG
**Number of sera**	N = 16	N = 16	N = 16	N = 16	N = 16	N = 16	N = 16	N = 16	N = 16	N = 16	N = 16	N = 16	N = 16	N = 16	N = 16	N = 16	N = 16	N = 16	N = 144	N = 144
**Antigens for ELISA**																				
EmVF	0	0	3	2	4	2	7	5	3	2	1	0	0	0	2	0	8	7	19.4%	12.5%
EmVC	0	0	4	3	4	3	4	4	1	0	3	0	2	0	4	4	5	4	18.8%	12.5%
EmP	0	0	3	3	1	2	0	0	0	0	0	0	0	0	3	3	2	4	6.3%	8.3%
Em2G11	0	0	1	1	5	4	6	3	4	2	2	1	7	3	7	6	9	9	28.5%	20.1%
mAb EmG3-EmVC	0	0	2	2	6	7	4	4	1	2	1	1	0	0	2	3	8	8	16.7%	18.8%
recEm18	0	0	0	0	4	4	1	1	0	0	0	0	0	0	0	0	3	3	5.6%	5.6%
recEm95	2	5	5	9	3	7	8	11	6	12	8	10	5	7	3	9	2	5	29.2%	52.1%
mAb EmG3-EgVC	0	0	4	4	5	5	7	7	1	1	1	1	0	0	7	7	7	7	22.2%	22.2%
EgVC	1	1	4	4	2	2	7	7	0	0	2	2	0	0	7	7	4	4	18.8%	18.8%
EgCF	0	0	8	7	9	8	7	5	9	2	0	0	0	0	7	5	15	15	38.2%	29.2%
EgP	0	0	8	7	4	3	4	3	1	0	0	0	0	0	5	4	10	8	22.2%	17.4%
EgAgB	0	0	4	4	8	9	7	7	8	10	0	0	0	0	9	11	15	15	35.4%	38.9%
recEg2B2	2	2	6	6	9	9	4	4	5	5	9	9	12	12	4	4	9	9	41.7%	41.7%
**Commercial test**																				
Western blot-species	13	13	1	1	2	2	0	0	0	0	0	0	0	0	0	0	0	0	11.1%	11.1%
Western blot-genus	1	1	1	1	3	3	3	3	0	0	0	0	0	0	1	1	5	5	9.7%	9.7%
EmVF-ELISA	0	0	0	0	6	6	5	5	2	2	0	0	0	0	5	5	8	8	18.1%	18.1%
EgP-IFAT	0	0	7	7	8	8	3	3	3	3	0	0	0	0	9	9	10	10	27.8%	27.8%
VectorBest-ELISA	0	0	1	1	3	3	4	4	0	0	0	0	0	0	3	3	12	12	16.0%	16.0%
Count of positive tests	19	22	62	62	86	87	81	76	44	41	27	24	26	22	78	81	132	133		

**Table 7:** Assays for the serological diagnosis of cystic echinococcosis—cross-reactions with 144 sera of patients with non-*Echinococcus* parasitic infections (Switzerland/CH, Kyrgyzstan/KG).

Parasitic Diseases (N = 144)	*Entamoeba*	*histolytica*	*Taenia*	*solium*	*Ascaris*	*lumbricoides*	*Trichinella*	spp.	*Strongyloides*	*stercoralis*	*Toxocara*	spp.	*Shistosoma*	spp.	*Fasciola*	*hepatica*	Filarial	Species	Total	Total
**Country**	CH	KG	CH	KG	CH	KG	CH	KG	CH	KG	CH	KG	CH	KG	CH	KG	CH	KG	CH	KG
**Number of sera**	N = 16	N = 16	N = 16	N = 16	N = 16	N = 16	N = 16	N = 16	N = 16	N = 16	N = 16	N = 16	N = 16	N = 16	N = 16	N = 16	N = 16	N = 16	N = 144	N = 144
**Antigens for ELISA**																				
EmVF	0	0	4	1	10	4	7	5	5	0	0	0	0	0	9	0	14	10	34.0%	13.9%
EmVC	5	2	6	5	7	6	6	5	6	5	3	3	4	2	7	6	7	6	35.4%	27.8%
EmP	3	3	6	6	5	5	2	2	10	10	2	2	3	3	3	3	4	4	26.4%	26.4%
mAb EmG3-EmVC	0	0	2	4	7	9	7	8	4	4	1	3	0	0	4	5	8	9	22.9%	29.1%
mAb EmG3-EgVC	0	0	6	5	7	7	8	8	3	2	2	1	2	0	8	7	9	9	31.3%	27.1%
EgVC	5	1	5	2	5	2	9	5	1	0	8	1	1	0	8	4	5	2	32.6%	11.8%
EgCF	0	0	7	7	8	8	9	5	11	2	1	0	0	0	11	5	15	15	43.1%	29.2%
EgP	0	0	8	7	7	3	7	3	4	0	0	0	0	0	8	4	12	8	31.9%	17.4%
EgAgB	0	0	4	4	9	8	7	6	11	8	0	0	0	0	11	8	15	15	39.6%	34.0%
recEg2B2	0	0	2	2	0	0	0	0	0	0	1	1	2	2	2	2	5	5	8.3%	8.3%
**Commercial test**																				
Western blot-species	13	13	1	1	2	2	0	0	0	0	0	0	0	0	0	0	0	0	11.1%	11.1%
Western blot-genus	1	1	1	1	3	3	3	3	0	0	0	0	0	0	1	1	5	5	9.7%	9.7%
EmVF-ELISA	0	0	0	0	6	6	5	5	2	2	0	0	0	0	5	5	8	8	18.1%	18.1%
EgP-IFAT	0	0	7	7	8	8	3	3	3	3	0	0	0	0	9	9	10	10	27.8%	27.8%
VectorBest-ELISA	0	0	1	1	3	3	4	4	0	0	0	0	0	0	3	3	12	12	16.0%	16.0%
Count of positive tests	27	20	60	53	87	74	77	62	60	36	18	11	12	7	89	62	129	118		

**Table 8:** Sensitivity and specificity of two-test combinations (ELISA) for the serological diagnosis of alveolar echinococcosis. The best test combinations for each group are marked in color (red for Switzerland, blue for Kyrgyzstan).

Test-Combinations		Sensitivity	Specificity	Sensitivity	Specificity
**(ELISA)**		(95% CI)	(95% CI)	(95% CI)	(95% CI)
		Switzerland	Switzerland	Kyrgyzstan	Kyrgyzstan
		AE * patients	blood donors	AE * patients	Ultrasound negative patients
Antigen 1	Antigen 2	N = 60	N = 68	N = 40	N = 68
mAb EmG3-EmVC	EmVF	**0.97 (0.88–0.99)**	**0.99 (0.92–0.99)**	0.70 (0.53–0.83)	0.90 (0.80–0.96)
mAb EmG3-EmVC	EmVC	**0.97 (0.88–0.99)**	**0.99 (0.92–0.99)**	0.68 (0.51–0.81)	0.85 (0.75–0.93)
mAb EmG3-EmVC	EmP	0.97 (0.88–0.99)	0.94 (0.86–0.98)	0.68 (0.51–0.81)	0.88 (0.78–0.95)
mAb EmG3-EmVC	Em2G11	0.97 (0.88–0.99)	0.97 (0.90–0.99)	0.75 (0.59–0.87)	0.83 (0.72–0.92)
mAb EmG3-EmVC	recEm18	0.97 (0.88–0.99)	0.94 (0.86–0.98)	0.66 (0.51–0.81)	0.81 (0.70–0.89)
mAb EmG3-EmVC	recEm95	0.97 (0.88–0.99)	0.76 (0.64–0.86)	**0.98 (0.87–0.99)**	**0.85 (0.75–0.93)**
mAb EmG3-EmVC	EgP	0.97 (0.90–0.99)	0.97 (0.90–0.99)	0.68 (0.51–0.81)	0.90 (0.73–0.92)
mAb EmG3-EmVC	EgCF	**0.99 (0.92–0.99)**	**0.99 (0.92–0.99)**	0.75 (0.59–0.87)	0.87 (0.76–0.94)
EmVF	EmVC	0.95 (0.86–0.99)	1.00 (0.94–1.00)	0.65 (0.48–0.79)	0.90 (0.80–0.96)
EmVF	EmP	0.95 (0.86–0.99)	0.96 (0.87–0.99)	0.73 (0.56–0.85)	0.90 (0.80–0.96)
EmVF	Em2G11	0.92 (0.82–0.97)	0.99 (0.92–0.99)	0.73 (0.56–0.85)	0.87 (0.76–0.94)
EmVF	recEm18	0.92 (0.82–0.97)	0.96 (0.87–0.99)	0.65 (0.48–0.79)	0.85 (0.75–0.93)
EmVF	recEm95	0.92 (0.82–0.97)	0.77 (0.66–0.87)	**0.93 (0.80–0.98)**	**0.90 (0.80–0.96)**
EmVF	EgP	0.92 (0.82–0.97)	0.99 (0.92–0.99)	0.68 (0.51–0.81)	0.91 (0.82–0.97)
EmVF	EgCF	**1.00 (0.94–1.00)**	**1.00 (0.95–1.00)**	0.70 (0.53–0.83)	0.88 (0.78–0.95)
EmP	EmVC	0.95 (0.86–0.99)	0.96 (0.87–0.99)	0.70 (0.53–0.83)	0.87 (0.76–0.94)
EmP	Em2G11	0.97 (0.88–0.99)	0.94 (0.86–0.98)	0.76 (0.61–0.89)	0.82 (0.71–0.91)
EmP	recEm18	0.97 (0.88–0.99)	0.91 (0.81–0.97)	0.70 (0.53–0.83)	0.82 (0.71–0.91)
EmP	recEm95	0.97 (0.88–0.99)	0.75 (0.63–0.85)	**0.98 (0.87–0.99)**	**0.87 (0.76–0.94)**
EmP	EgP	0.97 (0.88–0.99)	0.96 (0.88–0.99)	0.68 (0.51–0.81)	0.87 (0.76–0.94)
EmP	EgCF	0.97 (0.88–0.99)	0.96 (0.88–0.99)	0.78 (0.62–0.89)	0.84 (0.73–0.92)
Em2G11	EmVC	**0.97 (0.88–0.99)**	**0.99 (0.92–0.99)**	0.73 (0.56–0.85)	0.82 (0.71–0.91)
Em2G11	recEm18	0.92 (0.81–0.97)	0.94 (0.86–0.98)	0.73 (0.56–0.85)	0.76 (0.65–0.86)
Em2G11	recEm95	0.93 (0.84–0.98)	0.78 (0.66–0.87)	**0.98 (0.87–0.99)**	**0.81 (0.70–0.89)**
Em2G11	EgP	0.92 (0.82–0.97)	0.97 (0.90–0.99)	0.75 (0.59–0.87)	0.84 (0.73–0.92)
Em2G11	EgCF	0.92 (0.82–0.97)	0.99 (0.92–0.99)	0.73 (0.56–0.85)	0.84 (0.73–0.92)
recEm18	EmVC	0.97 (0.88–0.99)	0.96 (0.88–0.99)	0.65 (0.48–0.79)	0.82 (0.71–0.91)
recEm18	recEm95	0.90 (0.79–0.96)	0.76 (0.64–0.86)	**0.90 (0.76–0.97)**	**0.85 (0.75–0.93)**
recEm18	EgP	0.92 (0.82–0.97)	0.94 (0.86–0.98)	0.65 (0.48–0.79)	0.84 (0.73–0.92)
recEm18	EgCF	0.92 (0.82–0.97)	0.96 (0.88–0.99)	0.68 (0.51–0.81)	0.79 (0.68–0.88)
recEm95	EmVC	0.97 (0.88–0.99)	0.77 (0.66–0.87)	**0.95 (0.83–0.99)**	**0.85 (0.75–0.93)**
recEm95	EgP	0.93 (0.84–0.98)	0.76 (0.65–0.86)	**0.95 (0.83–0.99)**	**0.87 (0.76–0.94)**
recEm95	EgCF	0.93 (0.84–0.98)	0.78 (0.66–0.87)	**0.93 (0.80–0.98)**	**0.82 (0.71–0.91)**
EmVC	EgP	**0.97 (0.88–0.99)**	**0.99 (0.92–0.99)**	0.65 (0.34–0.79)	0.88 (0.78–0.95)
EmVC	EgCF	**0.97 (0.88–0.99)**	**1.00 (0.95–1.00)**	0.70 (0.53–0.83)	0.84 (0.73–0.92)
EgP	EgCF	0.92 (0.82–0.97)	0.99 (0.92–0.99)	0.73 (0.56–0.85)	0.88 (0.78–0.95)

* In countries where CE and AE co-exist, ultrasound is crucial for a correct diagnosis due to possible cross-reactions in diagnostic assays.

**Table 9:** Sensitivity and specificity of two-test combinations (ELISA) for the serological diagnosis of cystic echinococcosis. The best test combinations for each group are marked in color (red for Switzerland, blue for Kyrgyzstan).

Test-Combinations		Sensitivity	Specificity	Sensitivity	Specificity
**(ELISA)**		(95% CI)	(95% CI)	(95% CI)	(95% CI)
		Switzerland	Switzerland	Kyrgyzstan	Kyrgyzstan
		CE * patients	blood donors	CE * patients	Ultrasound negative patients
Antigen 1	Antigen 2	N = 41	N = 68	N = 23	N = 68
mAb EmG3-EgVC	EgCF	0.90 (0.77–0.97)	0.94 (0.86–0.98)	0.65 (0.43–0.84)	0.84 (0.73–0.92)
mAb EmG3-EgVC	EgP	0.93 (0.80–0.98)	0.94 (0.86–0.98)	0.74 (0.52–0.90)	0.85 (0.75–0.93)
mAb EmG3-EgVC	AgB	0.93 (0.80–0.98)	0.93 (0.84–0.98)	0.74 (0.52–0.90)	0.75 (0.63–0.85)
mAb EmG3-EgVC	recEg2B2	0.78 (0.62–0.89)	0.88 (0.78–0.95)	0.83 (0.61–0.95)	0.85 (0.75–0.93)
mAb EmG3-EgVC	EgVC	0.75 (0.60–0.88)	0.81 (0.70–0.89)	0.59 (0.36–0.79)	0.63 (0.51–0.75)
EgP	EgCF	**0.90 (0.77–0.97)**	**1.00 (0.95–1.00)**	0.74 (0.52–0.90)	0.87 (0.76–0.94)
EgP	AgB	**0.93 (0.80–0.98)**	**0.99 (0.92–0.99)**	0.78 (0.56–0.93)	0.78 (0.66–0.87)
EgP	recEg2B2	0.93 (0.80–0.98)	0.93 (0.84–0.98)	**0.87 (0.66–0.97)**	**0.89 (0.80–0.96)**
EgP	EgVC	0.93 (0.80–0.98)	0.84 (0.73–0.92)	0.74 (0.52–0.90)	0.63 (0.51–0.75)
AgB	EgCF	0.90 (0.77–0.97)	0.99 (0.92–0.99)	0.70 (0.47–0.87)	0.76 (0.65–0.86)
AgB	recEg2B2	0.93 (0.80–0.98)	0.91 (0.82–0.97)	0.83 (0.61–0.95)	0.79 (0.68–0.88)
AgB	EgVC	0.90 (0.77–0.97)	0.82 (0.71–0.91)	0.74 (0.52–0.90)	0.59 (0.46–0.71)
recEg2B2	EgCF	0.90 (0.77–0.97)	0.93 (0.84–0.98)	0.78 (0.56–0.93)	0.83 (0.73–0.92)
recEg2B2	EgVC	0.78 (0.62–0.89)	0.63 (0.51–0.75)	0.83 (0.61–0.95)	0.66 (0.54–0.77)
EgVC	EgCF	0.88 (0.73–0.96)	0.84 (0.73–0.92)	0.68 (0.45–0.86)	0.62 (0.49–0.73)

* In countries where CE and AE co-exist, ultrasound is crucial for a correct diagnosis due to possible cross-reactions in diagnostic assays.

## Data Availability

The data presented in this study is available on request from the corresponding authors. The data is not publicly available due to privacy reasons.

## Supplementary Materials

The following are available online at https://www.mdpi.com/article/10.3390/pathogens11050518/s1, Table S1: Swiss and Kyrgyz AE patients; overview sensitivity, specificity and cross-reactions, Table S2: Swiss and Kyrgyz CE patients; overview sensitivity, specificity and cross-reactions.
