# Supplementary material for: Serological Assays for Alveolar and Cystic Echinococcosis—A Comparative Multi-Test Study in Switzerland and Kyrgyzstan
Source: Pathogens. 2022 Apr 27;11(5):518. doi: 10.3390/pathogens11050518 (PMC9146094; doi:10.3390/pathogens11050518)
Supplement: Supplementary file 1 [file pathogens-11-00518-s001.zip › pathogens-1693483-supplementary.pdf]

Supplementary table S1. Swiss and Kyrgyz AE patients; overview sensitivity, specificity and cross-reactions

Antigens/Tests		Sensitivity		Specificity			Cross-reactions			
in house[1] and		Swiss	Kyrgyz	Swiss	Swiss	Kyrgyz	SwissCO	KyrgyzCO	SwissCO	KyrgyzCO
commercial[2]		AE patients		blood donors	NPLL	US negative	Parasites		CE patients	
N=23	Isolate	N=60	N=40	N=68	N=38	N=68	N=144		N=64	
EmVF*[1]	1. J2012	0.96 (0.86-0.99)	0.74 (0.54-0.90)	0.99 (0.93-0.99)	0.95 (0.82-0.99)	0.89 (0.76-0.97)	0.19 (0.13-0.28)	0.11 (0.09-0.12)	0.86 (0.75-0.93)	0.68 (0.56-0.79)
	2. AT17	0.95 (0.85-0.99)	0.77 (0.57-0.92)	0.98 (0.92-0.99)	0.95 (0.82-0.99)	0.92 (0.79-0.98)	0.26 (0.20-0.34)	0.19 (0.13-0.26)	0.88 (0.77-0.94)	0.74 (0.61-0.84)
EmVC*[1]	1. J2012	0.95 (0.85-0.99)	0.82 (0.63-0.95)	0.99 (0.94-0.99)	0.97 (0.86-0.99)	0.91 (0.79-0.98)	0.19 (0.13-0.26)	0.15 (0.10-0.17)	0.67 (0.54-0.78)	0.64 (0.51-0.76)
	2. AT17	0.96 (0.86-0.99)	0.79 (0.59-0.93)	0.99 (0.94-0.99)	0.97 (0.86-0.99)	0.91 (0.79-0.98)	0.26 (0.18-0.34)	0.18 (0.13-0.26)	0.81 (0.70-0.90)	0.70 (0.58-0.81)
EmP*[1]	1. J2012	0.96 (0.88-0.99)	0.82 (0.63-0.95)	0.98 (0.93-0.99)	0.95 (0.82-0.99)	0.95 (0.86-0.99)	0.08 (0.04-0.14)	0.10 (0.05-0.15)	0.59 (0.46-0.70)	0.61 (0.53-0.71)
	2. AT17	0.95 (0.85-0.99)	0.79 (0.59-0.93)	0.98 (0.92-0.99)	0.95 (0.82-0.99)	0.94 (0.83-0.99)	0.06 (0.03-0.11)	0.08 (0.04-0.14)	0.56 (0.43-0.69)	0.58 (0.45-0.70)
Em2G11*[1]	1. J2012	0.91 (0.80-0.98)	0.80 (0.62-0.94)	0.97 (0.88-0.99)	1.00 (0.91-1.00)	0.88 (0.76-0.97)	0.29 (0.22-0.37)	0.38 (0.23-0.43)	0.53 (0.40-0.66)	0.56 (0.43-0.69)
	2. AT17	0.91 (0.78-0.98)	0.78 (0.56-0.91)	0.96 (0.88-0.99)	1.00 (0.91-1.00)	0.87 (0.75-0.96)	0.29 (0.22-0.37)	0.38 (0.23-0.43)	0.53 (0.40-0.66)	0.55 (0.42-0.67)
mAb EmG3-EmVC*[1]	1. J2012	0.96 (0.87-0.99)	0.78 (0.59-0.93)	0.98 (0.91-0.99)	0.97 (0.86-0.99)	0.91 (0.79-0.98)	0.17 (0.11-0.24)	0.19 (0.13-0.26)	0.66 (0.53-0.77)	0.69 (0.56-0.80)
	2. AT17	0.94 (0.83-0.99)	0.75 (0.54-0.91)	0.97 (0.90-0.99)	0.92 (0.79-0.98)	0.89 (0.77-0.97)	0.11 (0.06-0.17)	0.11 (0.06-0.17)	0.72 (0.59-0.82)	0.72 (0.59-0.82)
recEm18[1]		0.82 (0.67-0.94)	0.70 (0.48-0.86)	0.96 (0.87-0.99)	0.95 (0.82-0.99)	0.89 (0.76-0.97)	0.06 (0.03-0.12)	0.06 (0.03-0.12)	0.13 (0.06-0.23)	0.13 (0.06-0.23)
recEm95[1]		0.67 (0.50-0.82)	0.82 (0.64-0.95)	0.91 (0.79-0.98)	0.92 (0.79-0.98)	0.95 (0.86-0.99)	0.28 (0.22-0.37)	0.52 (0.44-0.60)	0.14 (0.07-0.25)	0.25 (0.15-0.37)
mAb EmG3-EgVC[1]		0.78 (0.58-0.92)	0.78 (0.58-0.92)	0.83 (0.69-0.93)	0.86 (0.72-0.96)	0.83 (0.69-0.93)	0.22 (0.14-0.27)	0.22 (0.16-0.30)	0.66 (0.53-0.77)	0.66 (0.53-0.77)
EgVC[1]		0.75 (0.59-0.88)	0.73 (0.53-0.89)	0.83 (0.69-0.93)	0.92 (0.79-0.98)	0.67 (0.50-0.81)	0.19 (0.13-0.26)	0.22 (0.16-0.30)	0.53 (0.40-0.66)	0.41 (0.29-0.54)
EgCF[1]		0.95 (0.85-0.99)	0.76 (0.55-0.91)	0.99 (0.93-0.99)	0.97 (0.86-0.99)	0.88 (0.75-0.96)	0.38 (0.30-0.47)	0.29 (0.22-0.37)	0.86 (0.75-0.93)	0.73 (0.61-0.84)
EgP[1]		0.96 (0.87-0.99)	0.77 (0.57-0.92)	0.99 (0.93-0.99)	0.95 (0.82-0.99)	0.92 (0.81-0.98)	0.26 (0.18-0.29)	0.16 (0.11-0.24)	0.83 (0.71-0.91)	0.83 (0.71-0.91)
EgAgB[1]		0.89 (0.75-0.97)	0.70 (0.50-0.88)	0.98 (0.91-0.99)	0.95 (0.82-0.99)	0.81 (0.67-0.92)	0.35 (0.28-0.44)	0.40 (0.31-0.47)	0.83 (0.71-0.91)	0.84 (0.73-0.92)
recEg2B2[1]		0.64 (0.47-0.80)	0.65 (0.44-0.83)	0.64 (0.47-0.79)	0.84 (0.69-0.94)	0.65 (0.49-0.79)	0.41 (0.35-0.51)	0.40 (0.35-0.51)	0.58 (0.45-0.64)	0.84 (0.73-0.92)
Westernblot-genus[2]	EUROIMMUN	0.93 (0.84-0.97)	0.65 (0.50-0.78)	n/d	0.97 (0.86-0.99)	n/d	0.10 (0.06-0.14)	0.10 (0.06-0.14)	0.83 (0.71-0.91)	0.65 (0.53-0.76)
Westernblot-species[2]	EUROIMMUN	0.58 (0.46-0.70)	0.45 (0.31-0.60)	n/d	0.97 (0.86-0.99)	n/d	0.11 (0.07-0.15)	0.11 (0.07-0.15)	0.56 (0.43-0.69)	0.48 (0.37-0.60)
EmVF-ELISA[2]	EUROIMMUN	0.88 (0.78-0.94)	0.55 (0.40-0.69)	n/d	1.00 (0.91-1.00)	n/d	0.18 (0.12-0.25)	0.18 (0.12-0.25)	0.78 (0.67-0.87)	0.52 (0.40-0.63)
EgP-IFAT[2]	EUROIMMUN	0.90 (0.80-0.95)	0.68 (0.52-0.80)	n/d	0.97 (0.86-0.99)	n/d	0.28 (0.18-0.35)	0.28 (0.18-0.35)	0.83 (0.71-0.91)	0.74 (0.62-0.83)
VectorBest-ELISA[2]	VectorBest	0.77 (0.65-0.86)	0.45 (0.31-0.60)	n/d	1.00 (0.91-1.00)	n/d	0.16 (0.11-0.23)	0.16 (0.11-0.23)	0.63 (0.50-0.73)	0.35 (0.24-0.47)
*1. European and 2. Kyrgzy isolate				NPLL = non parasitic liver lesions			CO = Cut-Off		CO = Cut-Off	

Supplementary table S2. Swiss and Kyrgyz CE patients; overview sensitivity, specificity and cross-reactions

**Antigens/Tests**		**Sensitivity**		**Specificity**			**Cross-reactions**			
in house[1] and commercial[2]		Swiss CE patients	Kyrgyz CE patients	Swiss blood donors	Swiss NPLL	Kyrgyz US negative	SwissCO Parasites	KyrgyzCO Parasites	SwissCO AE patients	KyrgyzCO AE patients
N=19	Isolate	N=41	N=23	N=68	N=38	N=68	N=144		N=100	
EmVF*[1]	1. J2012	0.94 (0.80-0.99)	0.70 (0.42-0.90)	0.97 (0.89-0.99)	0.95 (0.82-0.99)	0.85 (0.72-0.94)	0.34 (0.26-0.42)	0.14 (0.09-0.21)	0.87 (0.79-0.93)	0.76 (0.66-0.84)
	2. AT17	0.91 (0.76-0.99)	0.76 (0.49-0.93)	0.95 (0.86-0.99)	0.95 (0.82-0.99)	0.85 (0.72-0.95)	0.40 (0.32-0.48)	0.29 (0.20-0.34)	0.79 (0.88-0.94)	0.81 (0.72-0.88)
EmVC*[1]	1. J2012	0.81 (0.63-0.95)	0.66 (0.38-0.89)	0.89 (0.98-0.99)	0.95 (0.82-0.99)	0.86 (0.73-0.95)	0.35 (0.28-0.44)	0.28 (0.21-0.36)	0.92 (0.85-0.96)	0.90 (0.82-0.95)
	2. AT17	0.89 (0.75-0.98)	0.70 (0.42-0.91)	0.97 (0.90-0.99)	0.97 (0.86-0.99)	0.86 (0.73-0.96)	0.33 (0.26-0.42)	0.22 (0.16-0.30)	0.90 (0.82-0.95)	0.86 (0.78-0.92)
EmP*[1]	1. J2012	0.84 (0.67-0.96)	0.76 (0.50-0.94)	0.96 (0.88-0.99)	0.92 (0.79-0.98)	0.94 (0.84-0.99)	0.26 (0.19-0.34)	0.26 (0.19-0.34)	0.83 (0.74-0.90)	0.83 (0.74-0.90)
	2. AT17	0.78 (0.59-0.93)	0.69 (0.41-0.89)	0.94 (0.84-0.99)	0.95 (0.82-0.99)	0.89 (0.75-0.96)	0.17 (0.11-0.24)	0.17 (0.11-0.24)	0.84 (0.75-0.91)	0.84 (0.75-0.91)
mAb EmG3-EmVC*[1]	1. J2012	0.84 (0.65-0.96)	0.67 (0.40-0.90)	0.93 (0.82-0.99)	0.97 (0.86-0.99)	0.88 (0.76-0.96)	0.23 (0.18-0.37)	0.29 (0.20-0.34)	0.88 (0.80-0.94)	0.86 (0.78-0.92)
	2. AT17	0.81 (0.62-0.94)	0.71 (0.44-0.91)	0.94 (0.84-0.99)	0.84 (0.69-0.94)	0.86 (0.73-0.95)	0.17 (0.11-0.24)	0.14 (0.09-0.21)	0.84 (0.75-0.91)	0.84 (0.75-0.91)
mAb EmG3-EgVC[1]		0.82 (0.63-0.95)	0.67 (0.40-0.89)	0.90 (0.78-0.95)	0.82 (0.66-0.92)	0.78 (0.63-0.90)	0.31 (0.24-0.39)	0.27 (0.20-0.35)	0.85 (0.76-0.91)	0.83 (0.74-0.90)
EgVC[1]		0.61 (0.40-0.80)	0.42 (0.17-0.69)	0.76 (0.61-0.89)	0.84 (0.69-0.94)	0.80 (0.66-0.91)	0.33 (0.21-0.36)	0.12 (0.07-0.16)	0.79 (0.70-0.87)	0.57 (0.47-0.67)
EgCF[1]		0.93 (0.79-0.99)	0.68 (0.40-0.90)	0.97 (0.90-0.99)	0.97 (0.86-0.99)	0.88 (0.75-0.96)	0.43 (0.35-0.55)	0.29 (0.20-0.34)	0.91 (0.84-0.96)	0.81 (0.72-0.88)
EgP[1]		0.93 (0.80-0.99)	0.76 (0.50-0.94)	0.97 (0.89-0.99)	0.92 (0.79-0.98)	0.93 (0.82-0.99)	0.32 (0.24-0.40)	0.17 (0.10-0.23)	0.86 (0.78-0.92)	0.78 (0.69-0.86)
EgAgB[1]		0.86 (0.69-0.97)	0.68 (0.42-0.90)	0.96 (0.86-0.99)	0.92 (0.79-0.98)	0.92 (0.79-0.98)	0.39 (0.32-0.48)	0.34 (0.26-0.42)	0.79 (0.70-0.87)	0.73 (0.63-0.81)
recEg2B2[1]		0.49 (0.29-0.70)	0.45 (0.19-0.72)	0.84 (0.71-0.95)	0.92 (0.79-0.98)	0.86 (0.73-0.95)	0.08 (0.04-0.14)	0.08 (0.04-0.14)	0.33 (0.24-0.43)	0.33 (0.24-0.43)
Westernblot-genus[2]	EUROIMMUN	0.83 (0.71-0.91)	0.65 (0.53-0.76)	n/d	0.97 (0.86-0.99)	n/d	0.10 (0.06-0.14)	0.10 (0.06-0.14)	0.93 (0.84-0.97)	0.65 (0.50-0.78)
Westernblot-species[2]	EUROIMMUN	0.56 (0.43-0.69)	0.48 (0.37-0.60)	n/d	0.97 (0.86-0.99)	n/d	0.11 (0.07-0.15)	0.11 (0.07-0.15)	0.58 (0.46-0.70)	0.45 (0.31-0.60)
EmVF-ELISA[2]	EUROIMMUN	0.78 (0.67-0.87)	0.52 (0.40-0.63)	n/d	1.00 (0.91-1.00)	n/d	0.18 (0.12-0.25)	0.18 (0.12-0.25)	0.88 (0.78-0.94)	0.55 (0.40-0.69)
EgP-IFAT[2]	EUROIMMUN	0.83 (0.71-0.91)	0.74 (0.62-0.83)	n/d	0.97 (0.86-0.99)	n/d	0.28 (0.18-0.35)	0.28 (0.18-0.35)	0.90 (0.80-0.95)	0.68 (0.52-0.80)
VectorBest-ELISA[2]	VectorBest	0.63 (0.50-0.73)	0.35 (0.24-0.47)	n/d	1.00 (0.91-1.00)	n/d	0.16 (0.11-0.23)	0.16 (0.11-0.23)	0.77 (0.65-0.86)	0.45 (0.31-0.60)

*1. European and 2. Kyrgzy isolate

NPLL = non parasitic liver lesions

CO = Cut-Off

CO = Cut-Off
